# Rotavirus Vaccination and the Global Burden of Rotavirus Diarrhea Among Children Younger Than 5 Years

**DOI:** 10.1001/jamapediatrics.2018.1960

**Published:** 2018-08-13

**Authors:** Christopher Troeger, Ibrahim A. Khalil, Puja C. Rao, Shujin Cao, Brigette F. Blacker, Tahmeed Ahmed, George Armah, Julie E. Bines, Thomas G. Brewer, Danny V. Colombara, Gagandeep Kang, Beth D. Kirkpatrick, Carl D. Kirkwood, Jason M. Mwenda, Umesh D. Parashar, William A. Petri, Mark S. Riddle, A. Duncan Steele, Robert L. Thompson, Judd L. Walson, John W. Sanders, Ali H. Mokdad, Christopher J. L. Murray, Simon I. Hay, Robert C. Reiner

**Affiliations:** 1 Institute for Health Metrics and Evaluation, Seattle, Washington; 2 International Centre for Diarrhoeal Disease Research, Dhaka, Bangladesh; 3 Noguchi Memorial Institute for Medical Research, Accra, Ghana; 4 Department of Paediatrics, University of Melbourne, Melbourne, Victoria, Australia; 5 Murdoch Children’s Research Institute, Department of Gastroenterology, Clinical Nutrition Royal Children's Hospital, Parkville, Melbourne, Victoria, Australia; 6 Global Enterics LLC, Seattle, Washington; 7 Translational Health Science and Technology Institute, Faridabad, India; 8 Department of Medicine, University of Vermont College of Medicine, Burlington; 9 Bill & Melinda Gates Foundation, Seattle, Washington; 10 World Health Organization Regional Office for Africa, Brazzaville, Republic of Congo; 11 Centers for Disease Control and Prevention, Atlanta, Georgia; 12 Division of Infectious Diseases and International Health, Department of Internal Medicine, University of Virginia, Charlottesville; 13 Uniformed Services University, Bethesda, Maryland; 14 Department of Global Health, University of Washington, Seattle; 15 Department of Medicine, University of Washington, Seattle; 16 Department of Pediatrics, University of Washington, Seattle; 17 Department of Epidemiology, University of Washington, Seattle; 18 Wake Forest University School of Medicine, Salem, North Carolina; 19 Big Data Institute, Li Ka Shing Centre for Health Information and Discovery, University of Oxford, Oxford, United Kingdom

## Abstract

**Importance:**

Rotavirus infection is the global leading cause of diarrhea-associated morbidity and mortality among children younger than 5 years.

**Objectives:**

To examine the extent of rotavirus infection among children younger than 5 years by country and the number of deaths averted because of the rotavirus vaccine.

**Design, Setting, and Participants:**

This report builds on findings from the Global Burden of Disease Study 2016, a cross-sectional study that measured diarrheal diseases and their etiologic agents. Models were used to estimate burden in data-sparse locations.

**Exposure:**

Diarrhea due to rotavirus infection.

**Main Outcomes and Measures:**

Rotavirus-associated mortality and morbidity by country and year and averted deaths attributable to the rotavirus vaccine by country.

**Results:**

Rotavirus infection was responsible for an estimated 128 500 deaths (95% uncertainty interval [UI], 104 500-155 600) among children younger than 5 years throughout the world in 2016, with 104 733 deaths occurring in sub-Saharan Africa (95% UI, 83 406-128 842). Rotavirus infection was responsible for more than 258 million episodes of diarrhea among children younger than 5 years in 2016 (95% UI, 193 million to 341 million), an incidence of 0.42 cases per child-year (95% UI, 0.30-0.53). Vaccine use is estimated to have averted more than 28 000 deaths (95% UI, 14 600-46 700) among children younger than 5 years, and expanded use of the rotavirus vaccine, particularly in sub-Saharan Africa, could have prevented approximately 20% of all deaths attributable to diarrhea among children younger than 5 years.

**Conclusions and Relevance:**

Rotavirus-associated mortality has decreased markedly over time in part because of the introduction of the rotavirus vaccine. This study suggests that prioritizing vaccine introduction and interventions to reduce diarrhea-associated morbidity and mortality is necessary in the continued global reduction of rotavirus infection.

## Key Points

**Questions:**

What is the extent of rotavirus infection among children younger than 5 years, and how has the rotavirus vaccine reduced this global burden?

**Findings:**

This report of the Global Burden of Disease and several extended analyses on rotavirus and results of rotavirus vaccination found that rotavirus infection caused 128 500 deaths and 258 173 300 episodes of diarrhea among children younger than 5 years in 2016. The rotavirus vaccine is estimated to have averted approximately 28 000 deaths in 2016, and approximately 83 200 additional children could have been saved had full vaccine coverage been achieved that year.

**Meaning:**

Prioritizing the introduction of the rotavirus vaccine and interventions to reduce diarrhea-associated morbidity and mortality are necessary in the continued reduction of the global rotavirus burden.

## Teaser

This analysis of data from the Global Burden of Disease Study examines the extent of rotavirus infection and associated deaths among children younger than 5 years worldwide and whether the rotavirus vaccine has reduced the diarrhea-associated mortality.

## Introduction

Diarrheal disease was the fourth leading cause of death among children younger than 5 years in 2015, responsible for nearly 500 000 deaths.^1,2^ Furthermore, rotavirus infection was responsible for 29.3% (95% uncertainty interval [UI], 24.6%-35.9%) of all diarrheal deaths among children younger than 5 years in 2015 (146 500 deaths; 95% UI, 118 000-183 500).^2^ Diarrhea-associated mortality has decreased since 1990 by nearly 65% in part because of improvements in safe water and sanitation and reductions in undernutrition among children younger than 5 years.^2^ Sustained efforts to scale and implement effective interventions must remain the long-term goal to alleviate the global diarrhea burden. In response to the urgent need to prevent avertable diarrhea episodes and their associated mortality, the World Health Organization (WHO) recommended that rotavirus vaccines be included in immunization programs in the European region and the Americas in 2006, a recommendation that was extended to all regions worldwide in 2009.^3^ In addition, Gavi, the Vaccine Alliance actively supports rotavirus vaccination by subsidizing the cost of vaccination in eligible countries.^3^

Rotavirus is 1 of the 13 diarrheal etiologic agents measured in the Global Burden of Disease Study 2016 (GBD 2016).^1,4,5^ The GBD 2016 is a systematic, scientific effort to produce comparable estimates of disease burden, including rotavirus diarrhea, for each age, sex, geographic location, and year from 1990 through 2016. This study describes the incidence and mortality of rotavirus infection among children younger than 5 years and reveals the urgent need for interventions to reduce diarrhea risk, treat severe diarrhea episodes, and prevent rotavirus diarrhea.

## Methods

Detailed methods on the GBD and diarrhea estimation in the GBD have been previously published.^1,4,5^ This report describes these methods briefly, focusing on etiologic attribution and changes from previous GBD methods. Intermediate models, input data, and code are available on the Global Health Data Exchange website.^6^ Institutional review board approval for the GBD 2016 was granted by the University of Washington.

Diarrhea-associated mortality was modeled in the Cause of Death Ensemble model (CODEm) platform. CODEm is a Bayesian, hierarchical, space-time, ensemble model tool.^2,7^ CODEm produces a wide variety of submodels designed to include a diverse set of covariates and model types. eTable 1 in the Supplement provides a complete list of the covariates used. Each submodel is weighted based on out-of-sample predictive validity and contributes draws to a final set of 1000 draws. These predictive regression models produce estimates of cause-specific mortality for each age, sex, geographic location, and year based on vital registration, verbal autopsy, and surveillance system data.

Diarrhea incidence was modeled in DisMod-MR, version 2.1 (DisMod). DisMod is a Bayesian, hierarchical metaregression tool.^5^ Like CODEm, DisMod uses space-time information and covariates to produce modeled estimates for each age, year, geographic location, and sex. DisMod also contains a compartmental model that enforces consistency among incidence, prevalence, and mortality. Input data are from the scientific literature, population-representative surveys, and health care use records.

Rotavirus was attributed to diarrhea-associated mortality and morbidity using a counterfactual approach called a population attributable fraction (PAF).^1^ This study’s approach accounted for pathogen codetection and detection in healthy individuals and does not necessitate a 1-pathogen–to–1-episode association. Detection of rotavirus was based on a molecular diagnostic case definition.^8^ The PAF is defined as follows^9^:

PAF = Proportion × (1 − 1/OR)

where OR is the odds ratio of diarrhea given pathogen detection and proportion is the modeled frequency of detection of rotavirus in diarrhea samples. The ORs are based on results from the Global Enteric Multicenter Study (GEMS) (eTable 2 in the Supplement).^8,10^ The proportion estimates are from DisMod models in which the input data are from scientific literature and modeled for each age, sex, year, and geographic location. Input data are given in eTable 3 and eFigure 1 in the Supplement. Results were adjusted to match a molecular diagnostic case definition using the sensitivity and specificity of enzyme-linked immunosorbent assay (ELISA) compared with the molecular diagnostic definition.

This study uses a systematic reanalysis of the GEMS samples using quantitative polymerase chain reaction (PCR).^8^ The modeling strategy requires that the continuous quantitative PCR test results are dichotomized into positive and negative results so that the ORs represent the strength of association between rotavirus and diarrhea.^11^ To do this, we identified the lowest cycle threshold in which the diagnostic accuracy was maximized (eFigure 2 in the Supplement). A summary of the input data for these models is provided in eTable 3 and eFigure 1 in the Supplement. To attribute diarrhea episodes and deaths due to rotavirus infection, the PAF estimates were multiplied by the diarrhea episode and death estimates. All estimates in the GBD 2016 are produced at the draw level with uncertainty carried through each step of the process, and values presented in this study are the mean values from these distributions, with UIs represented by the 2.5th and 97.5th percentiles of the distribution.

The ramifications of the rotavirus vaccine were evaluated using an analysis of the modeled rotavirus vaccine coverage. Rotavirus vaccine coverage was modeled using a space-time gaussian process regression in which the input data were the ratio of rotavirus vaccine coverage estimates from surveys and implementation programs to the coverage of 3 doses of diphtheria and tetanus toxoids and pertussis (DTP3) vaccine.^12^ Rotavirus vaccine coverage was modeled as a ratio to DTP3 coverage because it was assumed that DTP3 coverage was the upper bound of a routine immunization program’s ability to deliver the vaccine (eFigure 3 in the Supplement). A meta-analysis of the vaccine efficacy for severe diarrhea based on the reported values in a 2016 systematic review^13^ was used to estimate the relative reduction in rotavirus diarrhea associated with rotavirus vaccine use. The vaccine efficacy and the modeled vaccine coverage were used to estimate a counterfactual estimate of the rotavirus PAF in the absence of the vaccine by sex, year, and geographic location. The difference between the observed and a counterfactual estimate of the expected rotavirus PAF based on the vaccine coverage and efficacy was used to determine the reduction in deaths from diarrhea attributable to the vaccine.

## Results

**Figure 1.**
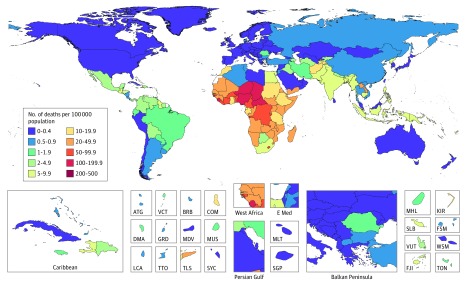
Geographic Distribution of Rotavirus-Associated Mortality Rates Among Children Younger Than 5 Years in 2016 ATG indicates Antigua; BRB, Barbados; COM, Comoros; DMA, Dominica; E Med, East Mediterranean; FJI, Fiji; FSM, Federated States of Micronesia; GRD, Grenada; KIR, Kiribati; LCA, Saint Lucia; MDV, Maldives; MHL, Marshall Islands; MLT, Malta; MUS, Mauritius; SGP, Singapore; SLB, Solomon Islands; SYC, Seychelles; TLS, Timor Leste; TON, Tongo; TTO, Trinidad and Tobago; VCT, Saint Vincent and the Grenadines; VUT, Vanuatu; and WSM, Samoa.

**Figure 2.**
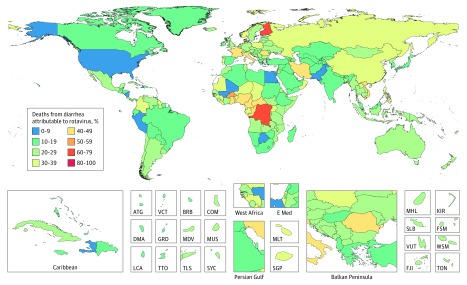
Distribution of the Fraction of Diarrhea-Associated Mortality Attributable to Rotavirus Among Children Younger Than 5 Years in 2016 ATG indicates Antigua; BRB, Barbados; COM, Comoros; DMA, Dominica; E Med, East Mediterranean; FJI, Fiji; FSM, Federated States of Micronesia; GRD, Grenada; KIR, Kiribati; LCA, Saint Lucia; MDV, Maldives; MHL, Marshall Islands; MLT, Malta; MUS, Mauritius; SGP, Singapore; SLB, Solomon Islands; SYC, Seychelles; TLS, Timor Leste; TON, Tongo; TTO, Trinidad and Tobago; VCT, Saint Vincent and the Grenadines; VUT, Vanuatu; and WSM, Samoa.

**Figure 3.**
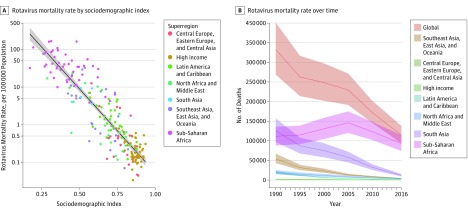
Sociodemographic and Spatial-Temporal Patterns in Rotavirus-Associated Mortality A, Rotavirus-associated mortality rate by sociodemographic index in 195 countries, estimated for 2016. B, Rotavirus mortality rate over time, globally and by superregion.

Rotavirus infection was responsible for an estimated 128 500 deaths (95% UI, 104 500-155 600) among children younger than 5 years in 2016; thus, 28.8% (95% UI, 25.0%-32.6%) of the deaths from diarrhea in this age group were attributable to rotavirus (Table). The rotavirus-associated mortality rate was highest in sub-Saharan Africa, Southeast Asia, and South Asia (Figure 1). A total of 104 733 deaths from rotavirus infection (95% UI, 83 406-128 842) among those younger than 5 years occurred in sub-Saharan Africa. Thirty percent of diarrhea deaths among children younger than 5 years were attributable to rotavirus infection in many geographic locations, seemingly independent of space or sociodemographic index (Figure 2 and Figure 3), with the attributable fraction caused by rotavirus ranging from 4.6% (95% UI, 3.0%-6.5%) in Nicaragua to 64.2% (95% UI, 61.0%-67.6%) in the Democratic Republic of the Congo (Figure 2 and eTable 4 in the Supplement). More than 50% of deaths from diarrhea in high-income countries, such as Denmark (51.0%; 95% UI, 44.8%-57.0%) and Finland (62.6%; 95% UI, 52.2%-73.0%), were attributable to rotavirus infection, indicating that it is a ubiquitous infection among children younger than 5 years.

**Table.** Burden of Rotavirus in 2016 Among Children Younger Than 5 Years by Global Burden of Disease Study Region and Superregion

Location	No. of Deaths (95% UI)	Mortality Rate, per 100 000 Population (95% UI)	Incidence, per 100 000 Population (95% UI)	No. of Cases (95% UI)
Global	128 530 (104 496-155 648)	20.3 (16.5-24.6)	401.3 (300.3-529.5)	258 173 278 (193 195 064-340 672 879)
Southeast Asia, East Asia, and Oceania	4499 (3498-5613)	3.7 (2.8-4.6)	305.5 (217.3-425.5)	38 563 330 (27 422 172-53 703 367)
East Asia	649 (478-880)	1.0 (0.7-1.4)	125.3 (85.4-183.7)	8 280 369 (5 643 598-12 134 956)
Southeast Asia	3765 (2895-4789)	6.6 (5.1-8.4)	507.9 (360.5-706.1)	29 845 328 (21 186 178-41 494 613)
Oceania	85 (44-153)	6.0 (3.1-10.8)	324.9 (208.3-487.1)	448 623 (287 666-672 555)
Central Europe, Eastern Europe, and Central Asia	317 (212-456)	1.1 (0.8-1.6)	431.1 (296.2-605.9)	13 107 287 (9 005 945-18 424 868)
Central Asia	237 (145-366)	2.5 (1.5-3.8)	172.5 (107.9-262.5)	1 899 833 (1 187 968-2 890 610)
Central Europe	24 (19-32)	0.4 (0.3-0.6)	722.6 (501.7-1 027.8)	4 125 508 (2 864 684-5 868 045)
Eastern Europe	55 (36-78)	0.4 (0.3-0.6)	520.4 (362.5-731.4)	7 122 181 (4 960 838-10 010 542)
High income	134 (109-164)	0.2 (0.2-0.3)	118.8 (81.0-171.7)	6 903 717 (4 707 088-9 978 464)
High-income Asia Pacific	10 (8-13)	0.1 (0.1-0.2)	33.0 (20.7-49.9)	251 204 (157 464-379 899)
Australasia	3 (2-4)	0.2 (0.1-0.2)	34.3 (21.7-52.3)	62 334 (39 402-94 978)
Western Europe	50 (38-64)	0.2 (0.2-0.3)	219.2 (143.9-327.8)	4 866 571 (3 196 026-7 279 975)
Southern Latin America	33 (25-43)	0.7 (0.5-0.8)	224.0 (166.3-301.5)	1 121 370 (832 556-1 509 433)
High-income North America	39 (29-50)	0.2 (0.1-0.2)	30.4 (19.2-45.2)	652 228 (412 958-970 517)
Latin America and Caribbean	1259 (976-1595)	2.5 (2.0-3.2)	447.0 (307.3-614.1)	21 138 621 (14 532 864-29 044 618)
Caribbean	198 (116-328)	5.0 (2.9-8.2)	186.6 (111.4-299.3)	770 044 (459 648-1 234 977)
Andean Latin America	80 (54-113)	1.2 (0.8-1.7)	256.1 (171.3-353.4)	1 679 785 (1 123 593-2 318 298)
Central Latin America	714 (538-930)	3.1 (2.4-4.1)	367.3 (241.6-533.6)	8 209 712 (5 400 527-11 925 432)
Tropical Latin America	267 (205-345)	1.7 (1.3-2.1)	740.1 (522.3-993.0)	10 553 252 (7 446 877-14 159 709)
North Africa and Middle East	4193 (2714-6422)	6.6 (4.3-10.2)	415.8 (267.4-608.8)	26 408 211 (16 981 918-38 665 090)
South Asia	13 396 (10 116-17 453)	8.7 (6.6-11.4)	220.1 (156.7-302.9)	35 169 025 (25 043 003-48 400 337)
Sub-Saharan Africa	104 733 (83 406-128 842)	66.9 (53.3-82.3)	742.6 (589.0-934.0)	117 303 716 (93 039 381-147 541 731)
Central sub-Saharan Africa	15 617 (9981-23 942)	75.1 (48.0-115.1)	1353.4 (1065.0-1683.2)	28 733 279 (22 610 651-35 735 936)
Eastern sub-Saharan Africa	16 300 (13 040-19 805)	26.1 (20.8-31.7)	432.1 (330.6-560.5)	26 952 299 (20 623 853-34 965 179)
Southern sub-Saharan Africa	1514 (1071-2071)	17.6 (12.4-24.1)	237.3 (157.5-342.2)	2 150 738 (1 427 096-3 101 783)
Western sub-Saharan Africa	71 303 (53 105-91 870)	110.3 (82.2-142.2)	915.2 (731.2-1151.4)	59 763 343 (47 747 505-75 187 780)

Abbreviation: UI, uncertainty interval.

Rotavirus infection was responsible for more than 258 million episodes of diarrhea among children younger than 5 years in 2016 (95% UI, 193 million to 341 million), an incidence of 0.42 cases per child-year (95% UI, 0.30-0.53) (Table and eTable 4 in the Supplement). The incidence among children younger than 5 years ranged from 0.024 cases per child-year (95% UI, 0.015-0.038) in South Korea to 1.63 cases per child-year (95% UI, 1.29-2.01) in the Democratic Republic of the Congo (Table and eTable 4 in the Supplement). Rotavirus was also an important cause of diarrhea morbidity in high-income locations, such as the United States, accounting for 593 000 episodes among children younger than 5 years (95% UI, 375 000-875 300) (country-level results are provided in eTable 4 in the Supplement). The estimated incidence of severe rotavirus diarrhea was 29.4 per 1000 child-years (95% UI, 22.2-38.0), accounting for 18 882 800 severe cases (95% UI, 14 263 600-24 446 700). Rotavirus infection was responsible for an estimated 1 537 000 (95% UI, 285 000-7 750 500) hospitalizations globally among children younger than 5 years in 2016. In addition, rotavirus-associated mortality among children younger than 5 years decreased by 48.2% (95% UI, 37.3-57.0) between 1990 and 2016 (Figure 3B).

**Figure 4.**
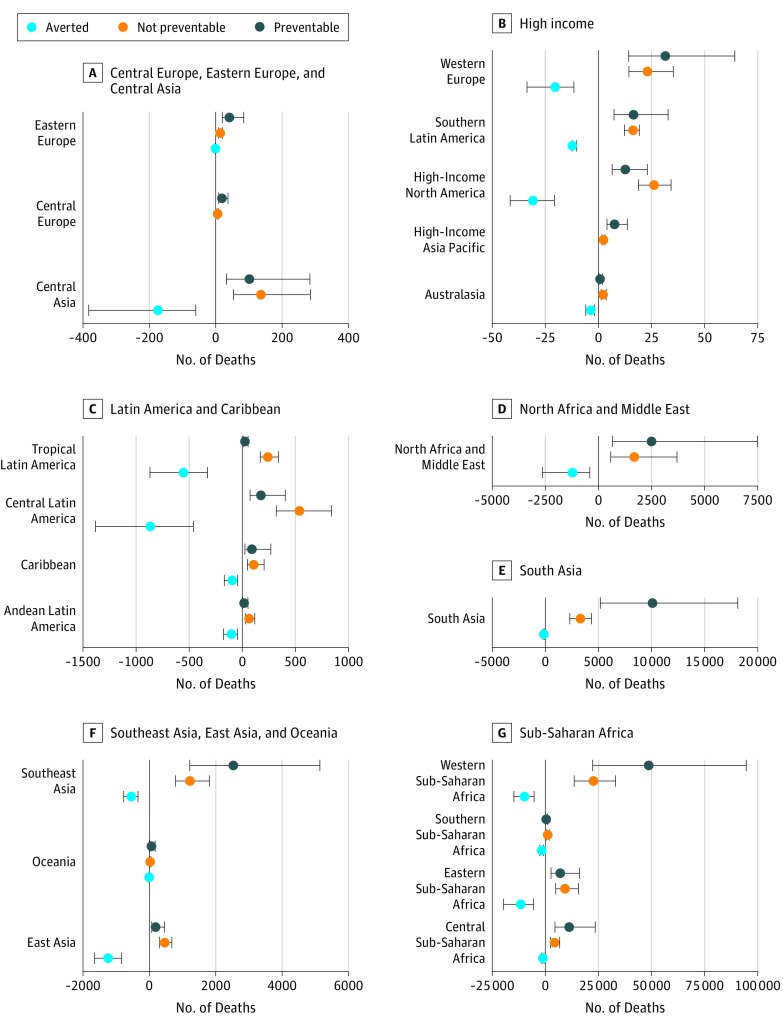
Number of Deaths Averted Because of Rotavirus Vaccine Coverage by Global Burden of Disease, Injuries, and Risk Factors Study Region Plots show the number of deaths among children younger than 5 years that were averted because of the rotavirus vaccine in 2016, the number of deaths that are potentially avertable given full coverage of the rotavirus vaccine, and the number of deaths that are not preventable given the current vaccine efficacy.

The introduction and expanded use of the rotavirus vaccine have already contributed to changes in rotavirus burden. This study estimated that 27.8% of children younger than 5 years were vaccinated against rotavirus in 2016 (eFigure 3 in the Supplement). The rotavirus vaccine averted an estimated 28 800 (95% UI, 14 600-46 700) deaths in 2016, including 24 200 (95% UI, 12 300-38 700) in sub-Saharan Africa, 1620 (95% UI, 870-2600) in Latin America, and 1410 (95% UI, 850-2300) in Southeast Asia, East Asia, and Oceania (Figure 4). The number of deaths averted depends on the fraction attributable to rotavirus infection and the vaccine effectiveness. This study estimated that full use of the rotavirus vaccine could have averted an additional 83 200 deaths (95% UI, 37 000-168 000) in 2016 (eTable 5 in the Supplement). Therefore, at the current coverage levels, only 15.3% of potentially avertable deaths were averted in 2016, and the regions with the highest rotavirus burden have not substantially averted deaths from rotavirus infection; full rotavirus vaccine coverage could avert 23% of all deaths due to diarrhea in sub-Saharan Africa (67 200 deaths; 95% UI, 29 600-135 300) and 10% of all deaths due to diarrhea in South Asia (10 100 deaths; 95% UI, 5200-18 100). Country-level results are given in eTable 5 and eFigure 4 in the Supplement. All results are available for further investigation on the Institute for Evaluation and Health Metrics website.^14^

## Discussion

### Rotavirus Burden

Although the burden of rotavirus has decreased during the past decade, rotavirus continues to be the leading cause of diarrhea-associated mortality among children younger than 5 years, responsible for nearly 130 000 deaths annually. Rotavirus was the third leading pathogen associated with mortality among those younger than 5 years in 2016, behind malaria (517 000 deaths) and *Streptococcus pneumoniae* (359 000 deaths).^4^ On average, more than 40% of children younger than 5 years had an episode of rotavirus diarrhea in 2016. However, there is a large discrepancy between the incidence and mortality of rotavirus infection in high- and low-income locations.

The number of deaths from diarrhea among children younger than 5 years has decreased by more than 45% since 2005.^4^ The changing mortality of rotavirus infection mirrors the large global decreases in diarrhea-associated mortality that have previously been attributed to improvements in water and sanitation and reductions in childhood undernutrition. However, rotavirus was estimated to be responsible for 7% of diarrhea episodes in those younger than 5 years in the United States and 10% in Canada, suggesting that rotavirus may be transmitted in pathways other than the fecal-oral route.^15^ The high transmissibility of rotavirus and difficulty in controlling it make widespread vaccine coverage a priority.

### Rotavirus Vaccine and Diarrheal Disease Burden 

By the end of 2015, Gavi, the Vaccine Alliance had funded rotavirus vaccine introductions in 37 countries and the Democratic Republic of the Congo, Bangladesh, and Nigeria, with introduction in Pakistan and India being planned in the near future,^16^ whereas 92 countries overall have introduced the vaccine.^17^ Recently developed vaccines, manufactured in India^18^ or China,^19^ will also likely contribute to more widespread use of rotavirus vaccine in these countries. Reductions in child deaths and hospitalizations from rotavirus diarrhea are expected after these introductions. Several countries that have implemented routine childhood vaccination against rotavirus have documented fewer cases of severe diarrhea and rotavirus disease requiring hospitalization^20^ and decreases of 22% to 50% in diarrhea-associated mortality.^21,22^ The present study showed that the vaccine has already alleviated the burden of diarrhea among those younger than 5 years in several countries that have introduced the vaccine, including Finland, Rwanda, and Ghana (eTable 5 and eFigure 4 in the Supplement). This study will provide decision makers with valuable information to develop strategies and achieve the international targets for alleviating the substantial burden of diarrheal diseases.

### Comparison With Other Estimates

The GBD is on an annual publication cycle in which the number of deaths due to rotavirus diarrhea and deaths due to all causes is reestimated every year, allowing for incremental improvements to the rotavirus burden estimation strategy. A comparison of estimates between GBD 2015 and 2016 is shown in eFigure 5 in the Supplement. The number of deaths from rotavirus infection among children younger than 5 years is similar between GBD 2016 (128 500; 95% UI, 104 500-155 600) and GBD 2015 (146 000; 95% UI, 118 000-183 000).^1,4^

A recent publication^23^ made comprehensive comparisons between the GBD 2013 estimates for rotavirus-associated mortality and those produced by the Child Health Epidemiology Research Group (CHERG), the WHO, and the Centers for Disease Control and Prevention (CDC). The number of deaths from rotavirus infection in the GBD 2016 is lower than the estimates produced by the other groups. The WHO and CDC estimated 215 757 deaths in 2013, and CHERG estimated 157 398 in 2013.^23,24,25^ In contrast, the GBD 2016 estimated 193 300 deaths in 2010 (95% UI, 163 800-227 500) and 128 500 deaths (95% UI, 104 500-155 600) in 2016. Each set of estimates used different approaches, input data, and model assumptions to reach their conclusions. The CHERG as well as WHO and CDC groups used the same estimate of diarrhea-associated mortality among those younger than 5 years, which is marginally larger than that produced by the GBD.^23^

### Limitations

Data on the incidence of diarrhea and its causes are scarce—an issue that is often most prevalent in high-burden locations. Subsequently, estimates in these locations rely more heavily on models based on predictive validity. To address this issue of sparse mortality and etiologic data, we propagated uncertainty through every step of the modeling process (eFigure 6 in the Supplement). In addition, this study addressed the association among the rotavirus vaccine, hospitalizations, and burden but is limited by the fact that hospitalization in many countries is not a function of severity but of access to care.^26^ In regions such as these, when hospital access is not restricted, hospitalization rates are much higher. There may be a shift in the use of diagnostics for rotavirus from ELISA to molecular diagnostics, particularly for surveillance and clinical trials.^27,28,29^ Although the sensitivity and specificity of ELISA diagnostics to molecular diagnostic detection frequency are high (>95%), there may be a detection bias among studies included in this analysis, particularly if there are strong time or geographic trends in the types of diagnostics used. The use of molecular diagnostics as a criterion standard and the association between ELISA and quantitative PCR used in the present study was informed by a single case-control study (GEMS).^8,10^ In addition, because GEMS was a study of moderate to severe diarrhea, this study makes the assumption that the cause of severe diarrhea is comparable with that of fatal diarrhea.

## Conclusions

These results suggest that rotavirus is still a highly prevalent cause of diarrhea worldwide and is responsible for a substantial nonfatal burden of diarrhea globally. These findings call for renewed efforts to prevent rotavirus infection through increased efforts to improve vaccination coverage, water and sanitation, and access to and quality of medical care. Governments, donors, and health care professionals should use these findings to reduce the burden of rotavirus at the country, regional, and global levels.

## Notes

Supplement. eTable 1. Covariates used in GBD 2016 diarrhea modeling eTable 2. The association between rotavirus detection and diarrhea case status eTable 3. Data points used for rotavirus proportion modeling by country and source population eTable 4. Deaths, mortality rate per 100,000, incidence per 1000, and number of cases due to rotavirus in 2016 among children under 5 eTable 5. Deaths averted due to the rotavirus vaccine and remaining avertable deaths if vaccine coverage were 100% in 2016 by GBD region and country eFigure 1. Data coverage maps eFigure 2. Diagnostic ability to discriminate cases and controls of qPCR eFigure 3. Modeled rotavirus vaccine coverage among children under 5 in 2016 eFigure 4. Modeled number of under 5 deaths averted due to rotavirus vaccine in 2016 eFigure 5. A comparison of the number of deaths and PAFs due to rotaviral diarrhea in GBD 2015 and GBD 2016 in all ages and children under-5 eFigure 6. Visualizing uncertainty around PAF estimates

## References

[1] GBD Diarrhoeal Diseases Collaborators Estimates of global, regional, and national morbidity, mortality, and aetiologies of diarrhoeal diseases: a systematic analysis for the Global Burden of Disease Study 2015. Lancet Infect Dis. 2017;17(9):909-948. doi:10.1016/S1473-3099(17)30276-1 28579426 PMC5589208

[2] GBD 2015 Mortality and Causes of Death Collaborators Global, regional, and national life expectancy, all-cause mortality, and cause-specific mortality for 249 causes of death, 1980-2015: a systematic analysis for the Global Burden of Disease Study 2015. Lancet. 2016;388(10053):1459-1544. doi:10.1016/S0140-6736(16)31012-1 27733281 PMC5388903

[3] World Health Organization Rotavirus vaccines: an update. Wkly Epidemiol Rec. 2009;84(50):533-540. 20034143

[4] GBD 2016 Causes of Death Collaborators Global, regional, and national age-sex specific mortality for 264 causes of death, 1980-2016: a systematic analysis for the Global Burden of Disease Study 2016. Lancet. 2017;390(10100):1151-1210. doi:10.1016/S0140-6736(17)32152-9 28919116 PMC5605883

[5] GBD 2016 Disease and Injury Incidence and Prevalence Collaborators Global, regional, and national incidence, prevalence, and years lived with disability for 328 diseases and injuries for 195 countries, 1990-2016: a systematic analysis for the Global Burden of Disease Study 2016. Lancet. 2017;390(10100):1211-1259. doi:10.1016/S0140-6736(17)32154-2 28919117 PMC5605509

[6] Global Health Data Exchange | GHDx. http://ghdx.healthdata.org/. Accessed June 8, 2018.

[7] Foreman KJ, Lozano R, Lopez AD, Murray CJ Modeling causes of death: an integrated approach using CODEm. Popul Health Metr. 2012;10:1. doi:10.1186/1478-7954-10-1 22226226 PMC3315398

[8] Liu J, Platts-Mills JA, Juma J, Use of quantitative molecular diagnostic methods to identify causes of diarrhoea in children: a reanalysis of the GEMS case-control study. Lancet. 2016;388(10051):1291-1301. doi:10.1016/S0140-6736(16)31529-X 27673470 PMC5471845

[9] Miettinen OS Proportion of disease caused or prevented by a given exposure, trait or intervention. Am J Epidemiol. 1974;99(5):325-332. doi:10.1093/oxfordjournals.aje.a121617 4825599

[10] Kotloff KL, Nataro JP, Blackwelder WC, Burden and aetiology of diarrhoeal disease in infants and young children in developing countries (the Global Enteric Multicenter Study, GEMS): a prospective, case-control study. Lancet. 2013;382(9888):209-222. doi:10.1016/S0140-6736(13)60844-2 23680352

[11] Liu J, Gratz J, Amour C, Optimization of quantitative PCR methods for enteropathogen detection. PLoS One. 2016;11(6):e0158199. doi:10.1371/journal.pone.0158199 27336160 PMC4918952

[12] GBD 2016 Risk Factors Collaborators Global, regional, and national comparative risk assessment of 84 behavioural, environmental and occupational, and metabolic risks or clusters of risks, 1990-2016: a systematic analysis for the Global Burden of Disease Study 2016. Lancet. 2017;390(10100):1345-1422. doi:10.1016/S0140-6736(17)32366-8 28919119 PMC5614451

[13] Lamberti LM, Ashraf S, Walker CLF, Black RE A systematic review of the effect of rotavirus vaccination on diarrhea outcomes among children younger than 5 years. Pediatr Infect Dis J. 2016;35(9):992-998. doi:10.1097/INF.0000000000001232 27254030

[14] GBD Compare | IHME Viz Hub. http://vizhub.healthdata.org/gbd-compare. Accessed June 8, 2018.

[15] Chen S-C, Tan L-B, Huang L-M, Chen K-T Rotavirus infection and the current status of rotavirus vaccines. J Formos Med Assoc. 2012;111(4):183-193. doi:10.1016/j.jfma.2011.09.024 22526206

[16] Gavi, the Vaccine Alliance. Rotavirus vaccine support. http://www.gavi.org/support/nvs/rotavirus/. Accessed November 15, 2017.

[17] Rota Council Vaccine introduction. http://rotacouncil.org/vaccine-introduction/. Accessed November 15, 2017.

[18] Chandola TR, Taneja S, Goyal N, ROTAVAC^®^ does not interfere with the immune response to childhood vaccines in Indian infants: a randomized placebo controlled trial. Heliyon. 2017;3(5):e00302. doi:10.1016/j.heliyon.2017.e00302 28560356 PMC5435614

[19] Zhen S-S, Li Y, Wang S-M, Effectiveness of the live attenuated rotavirus vaccine produced by a domestic manufacturer in China studied using a population-based case-control design. Emerg Microbes Infect. 2015;4(10):e64. doi:10.1038/emi.2015.64 26576341 PMC4631931

[20] Patel MM, Glass R, Desai R, Tate JE, Parashar UD Fulfilling the promise of rotavirus vaccines: how far have we come since licensure? Lancet Infect Dis. 2012;12(7):561-570. doi:10.1016/S1473-3099(12)70029-4 22742639

[21] Paternina-Caicedo A, Parashar UD, Alvis-Guzmán N, Effect of rotavirus vaccine on childhood diarrhea mortality in five Latin American countries. Vaccine. 2015;33(32):3923-3928. doi:10.1016/j.vaccine.2015.06.058 26116247 PMC11285373

[22] Richardson V, Hernandez-Pichardo J, Quintanar-Solares M, Effect of rotavirus vaccination on death from childhood diarrhea in Mexico. N Engl J Med. 2010;362(4):299-305. doi:10.1056/NEJMoa0905211 20107215

[23] Clark A, Black R, Tate J, ; Global Rotavirus Surveillance Network Estimating global, regional and national rotavirus deaths in children aged <5 years: current approaches, new analyses and proposed improvements. PLoS One. 2017;12(9):e0183392. doi:10.1371/journal.pone.0183392 28892480 PMC5593200

[24] World Health Organization Estimated rotavirus deaths for children under 5 years of age: 2013, 215 000. WHO. http://www.who.int/immunization/monitoring_surveillance/burden/estimates/rotavirus/en/. Accessed November 15, 2017.

[25] Lanata CF, Fischer-Walker CL, Olascoaga AC, Torres CX, Aryee MJ, Black RE; Child Health Epidemiology Reference Group of the World Health Organization and UNICEF Global causes of diarrheal disease mortality in children <5 years of age: a systematic review. PLoS One. 2013;8(9):e72788. doi:10.1371/journal.pone.0072788 24023773 PMC3762858

[26] John J, Sarkar R, Muliyil J, Bhandari N, Bhan MK, Kang G Rotavirus gastroenteritis in India, 2011-2013: revised estimates of disease burden and potential impact of vaccines. Vaccine. 2014;32(suppl 1):A5-A9. doi:10.1016/j.vaccine.2014.03.004 25091681

[27] Platts-Mills JA, Operario DJ, Houpt ER Molecular diagnosis of diarrhea: current status and future potential. Curr Infect Dis Rep. 2012;14(1):41-46. doi:10.1007/s11908-011-0223-7 22116640 PMC3253426

[28] Platts-Mills JA, Babji S, Bodhidatta L, ; MAL-ED Network Investigators Pathogen-specific burdens of community diarrhoea in developing countries: a multisite birth cohort study (MAL-ED). Lancet Glob Health. 2015;3(9):e564-e575. doi:10.1016/S2214-109X(15)00151-5 26202075 PMC7328884

[29] Operario DJ, Platts-Mills JA, Nadan S, Etiology of severe acute watery diarrhea in children in the global rotavirus surveillance network using quantitative polymerase chain reaction. J Infect Dis. 2017;216(2):220-227. doi:10.1093/infdis/jix294 28838152 PMC5853801

