# Supplementary material for: Rotavirus Vaccination and the Global Burden of Rotavirus Diarrhea Among Children Younger Than 5 Years
Source: JAMA Pediatr. 2018 Aug 13;172(10):958–65. doi: 10.1001/jamapediatrics.2018.1960 (PMC6233802; doi:10.1001/jamapediatrics.2018.1960)
Supplement: Supplement. — eTable 1. Covariates used in GBD 2016 diarrhea modeling eTable 2. The association between rotavirus detection and diarrhea case status eTable 3. Data points used for rotavirus proportion modeling by country and source population eTable 4. Deaths, mortality rate per 100,000, incidence per 1000, and number of cases due to rotavirus in 2016 among children under 5 eTable 5. Deaths averted due to the rotavirus vaccine and remaining avertable deaths if vaccine coverage were 100% in 2016 by GBD region and country eFigure 1. Data coverage maps eFigure 2. Diagnostic ability to discriminate cases and controls of qPCR eFigure 3. Modeled rotavirus vaccine coverage among children under 5 in 2016 eFigure 4. Modeled number of under 5 deaths averted due to rotavirus vaccine in 2016 eFigure 5. A comparison of the number of deaths and PAFs due to rotaviral diarrhea in GBD 2015 and GBD 2016 in all ages and children under-5 eFigure 6. Visualizing uncertainty around PAF estimates [file jamapediatr-172-958-s001.pdf]

Supplementary Online Content

Troeger C, Khalil IA, Rao PC, et al. Rotavirus Vaccination and the Global Burden of Rotavirus Diarrhea Among Children Younger Than 5 Years. *JAMA Pediatr.* Published online August 13, 2018. doi:10.1001/jamapediatrics.2018.1960

Supplement. eTable 1. Covariates used in GBD 2016 diarrhea modeling

eTable 2. The association between rotavirus detection and diarrhea case status

eTable 3. Data points used for rotavirus proportion modeling by country and source population

eTable 4. Deaths, mortality rate per 100,000, incidence per 1000, and number of cases due to rotavirus in 2016 among children under 5

eTable 5. Deaths averted due to the rotavirus vaccine and remaining avertable deaths if vaccine coverage were 100% in 2016 by GBD region and country

eFigure 1. Data coverage maps

eFigure 2. Diagnostic ability to discriminate cases and controls of qPCR

eFigure 3. Modeled rotavirus vaccine coverage among children under 5 in 2016

eFigure 4. Modeled number of under 5 deaths averted due to rotavirus vaccine in 2016

eFigure 5. A comparison of the number of deaths and PAFs due to rotaviral diarrhea in GBD 2015 and GBD 2016 in all ages and children under-5

eFigure 6. Visualizing uncertainty around PAF estimates

This supplementary material has been provided by the authors to give readers additional information about their work.

eTable 1. Covariates used in GBD 2016 diarrhea modeling.

Level is ranked from 1 (causally related) to 3 (distally related). Direction is the required negative or positive association with diarrhea mortality.

Covariate	Level	Direction
Diarrhea Summary Exposure Variable (SEV)	1	+
Height for age <2 SD	1	+
Sanitation SEV	1	+

Water SEV	1	+
Weight for age <2 SD	1	+
Weight for height <2 SD	1	+
Handwashing	1	-
Rotavirus vaccine	1	-
Safe sanitation	1	-
Safe water	1	-
Breastfeeding SEV	2	+
Vitamin A deficiency	2	+
Zinc deficiency	2	+
Healthcare access and quality index	2	-
LDI per Capita	3	-
Maternal education per capita	3	-
Socio-demographic index	3	-
Population <150/km2	3	0
Population >1000/km2	3	0

© 2018 Troeger C et al. *JAMA Pediatrics.*

eTable 2. The association between rotavirus detection and diarrhea case status.

The odds ratios of diarrhea given the presence of rotavirus in a stool sample, based on a molecular diagnostic case definition, is shown for the two age groups used in this study.

Age	Odds ratio (95% CI)
Under 1	15.4 (9.9-23.1)
Over 1	14.1 (7.4-25.7)

© 2018 Troeger C et al. *JAMA Pediatrics.*

eTable 3. Data points used for rotavirus proportion modeling by country and source population.

Location	iso3 Code	Data points	Inpatient population	Rotavirus exclusive testing	New for GBD 2016
Angola	AGO	2	1	0	2
Albania	ALB	6	5	0	6
Argentina	ARG	22	19	22	0
Australia	AUS	24	9	0	0
Austria	AUT	2	0	0	1
Belgium	BEL	14	14	7	0
Burkina Faso	BFA	17	8	5	5
Bangladesh	BGD	98	48	41	1
Bulgaria	BGR	8	8	0	0
Bosnia and Herzegovina	BIH	5	5	0	1
Bolivia	BOL	3	3	0	2
Brazil	BRA	141	93	87	6
Botswana	BWA	17	10	12	0
Central African Republic	CAF	11	7	8	3
Switzerland	CHE	1	1	0	1
Chile	CHL	12	9	0	0
China	CHN	94	42	29	12
Cote d'Ivoire	CIV	4	4	4	0
Cameroon	CMR	29	7	15	0
Democratic Republic of the Congo	COD	11	0	6	0
Congo	COG	7	0	7	0
Colombia	COL	8	0	0	4
Cuba	CUB	4	4	4	0
Germany	DEU	20	16	14	1
Denmark	DNK	3	0	3	0
Ecuador	ECU	1	0	0	0
Egypt	EGY	20	1	0	0
England	ENG	3	0	0	0
Spain	ESP	85	74	47	1
Estonia	EST	3	3	2	0
Ethiopia	ETH	9	8	8	0
Finland	FIN	7	3	0	1
Fiji	FJI	9	9	0	0
France	FRA	16	14	7	1
Gabon	GAB	2	0	0	2
Ghana	GHA	42	1	17	1
The Gambia	GMB	21	9	6	0

© 2018 Troeger C et al. *JAMA Pediatrics.*

Location	iso3 Code	Data points	Inpatient population	Rotavirus exclusive testing	New for GBD 2016
Guinea-Bissau	GNB	5	5	5	0
Greece	GRC	8	2	5	1
Guatemala	GTM	25	10	8	3
Guyana	GUY	1	1	0	0
Honduras	HND	2	1	0	0
Haiti	HTI	11	11	0	0
Hungary	HUN	13	13	13	0
Indonesia	IDN	30	19	8	7
India	IND	191	87	146	48
Ireland	IRL	1	0	0	1
Iran	IRN	7	6	0	0
Iceland	ISL	7	0	0	0
Israel	ISR	3	2	2	0
Italy	ITA	32	23	7	3
Jordan	JOR	2	2	0	0
Japan	JPN	25	2	16	7
Kazakhstan	KAZ	13	13	13	0
Kenya	KEN	88	18	58	0
Kyrgyzstan	KGZ	21	21	15	0
Cambodia	KHM	9	8	7	0
South Korea	KOR	54	48	45	0
Laos	LAO	8	5	0	0
Libya	LBY	5	4	0	2
Sri Lanka	LKA	1	1	0	1
Morocco	MAR	9	9	5	0
Madagascar	MDG	8	1	8	0
Mali	MLI	17	9	1	0
Mongolia	MNG	2	2	0	2
Mozambique	MOZ	18	9	0	3
Mauritius	MUS	7	0	7	0
Malawi	MWI	4	1	3	0
Malaysia	MYS	2	1	1	0
Namibia	NAM	10	0	10	10
Niger	NER	1	0	0	0
Nigeria	NGA	43	2	32	23
Nicaragua	NIC	12	0	1	3
Netherlands	NLD	5	5	0	0
Nepal	NPL	65	18	32	1
Oman	OMN	2	2	0	0

© 2018 Troeger C et al. *JAMA Pediatrics.*

Location	iso3 Code	Data points	Inpatient population	Rotavirus exclusive testing	New for GBD 2016
Pakistan	PAK	33	24	15	0
Peru	PER	22	7	3	3
Philippines	PHL	9	9	8	0
Papua New Guinea	PNG	6	6	0	0
Poland	POL	12	2	6	0
Portugal	PRT	1	0	0	1
Paraguay	PRY	19	19	18	0
Western Europe	R10	3	0	0	3
Southern Sub-Saharan Africa	R20	1	0	1	0
Romania	ROU	4	3	0	1
Russia	RUS	7	7	0	0
Rwanda	RWA	1	0	0	0
Saudi Arabia	SAU	8	3	4	1
Sudan	SDN	4	0	1	0
Senegal	SEN	4	1	0	0
Singapore	SGP	10	9	0	1
El Salvador	SLV	3	3	1	0
Suriname	SUR	1	1	0	0
Sweden	SWE	8	8	7	0
Togo	TGO	1	0	1	0
Thailand	THA	39	18	2	1
Tunisia	TUN	16	15	13	0
Turkey	TUR	20	13	6	4
Taiwan	TWN	2	2	0	0
Tanzania	TZA	7	0	1	1
Uganda	UGA	25	24	6	6
North East England	UKC	16	16	0	0
North West England	UKD	25	24	8	0
Yorkshire and the Humber	UKE	18	18	0	0
East Midlands	UKF	11	11	0	0
West Midlands	UKG	18	17	0	0
East of England	UKH	16	16	0	0
Greater London	UKI	18	18	0	0
South East England	UKJ	12	12	0	0
South West England	UKK	14	14	0	0
United States	USA	26	9	0	8
Uzbekistan	UZB	18	18	15	0
Saint Vincent and the Grenadines	VCT	1	1	0	0
Venezuela	VEN	15	4	7	0

© 2018 Troeger C et al. *JAMA Pediatrics.*

Location	iso3 Code	Data points	Inpatient population	Rotavirus exclusive testing	New for GBD 2016
Vietnam	VNM	27	21	7	0
South Africa	ZAF	19	15	16	0
Zambia	ZMB	10	9	9	9
Zimbabwe	ZWE	13	12	12	0

© 2018 Troeger C et al. *JAMA Pediatrics.*

eTable 4. Deaths, mortality rate per 100,000, incidence per 1000, and number of cases due to rotavirus in 2016 among children under 5.

Location	Deaths (95% UI)	Mortality rate per 100,000 (95% UI)	Incidence per 1,000 (95% UI)	Cases (95% UI)
**Global**	**128,530 (104,496-155,648)**	**20.3 (16.5-24.6)**	**401.3 (300.3-529.5)**	**258,173,278 (193,195,064-340,672,879)**
**Southeast Asia, East Asia, and Oceania**	**4,499 (3,498-5,613)**	**3.7 (2.8-4.6)**	**305.5 (217.3-425.5)**	**38,563,330 (27,422,172-53,703,367)**
**East Asia**	**649 (478-880)**	**1.0 (0.7-1.4)**	**125.3 (85.4-183.7)**	**8,280,369 (5,643,598-12,134,956)**
China	474 (363-621)	0.8 (0.6-1.0)	111.1 (76.7-161.2)	6,862,616 (4,736,201-9,951,575)
North Korea	173 (82-310)	5.8 (2.8-10.5)	370.0 (235.0-546.0)	1,214,987 (771,802-1,792,896)
Taiwan	2 (1-3)	0.2 (0.1-0.3)	194.3 (116.3-304.4)	202,885 (121,429-317,854)
**Southeast Asia**	3,765 (2,895-4,789)	6.6 (5.1-8.4)	507.9 (360.5-706.1)	29,845,328 (21,186,178-41,494,613)
Cambodia	96 (55-158)	5.1 (2.9-8.4)	667.9 (453.8-921.4)	1,223,928 (831,622-1,688,584)
Indonesia	1,994 (1,498-2,611)	8.8 (6.6-11.5)	538.7 (369.3-776.0)	12,948,711 (8,878,371-18,654,115)
Laos	329 (139-622)	29.1 (12.3-55.0)	833.1 (569.3-1,158.0)	899,194 (614,471-1,249,885)
Malaysia	7 (5-12)	0.3 (0.2-0.5)	222.5 (126.0-357.7)	579,057 (328,009-931,153)
Maldives	0 (0-0)	0.4 (0.3-0.7)	258.1 (164.9-396.8)	9,512 (6,076-14,621)
Mauritius	1 (1-2)	1.7 (1.1-2.5)	374.8 (240.1-574.2)	24,854 (15,923-38,074)
Myanmar	324 (148-609)	7.0 (3.2-13.1)	334.7 (214.7-499.9)	2,093,714 (1,343,046-3,127,199)
Philippines	887 (558-1,382)	7.7 (4.8-11.9)	507.3 (390.2-671.1)	5,868,418 (4,514,026-7,763,542)
Sri Lanka	3 (2-6)	0.2 (0.1-0.4)	234.7 (147.8-358.6)	326,929 (205,905-499,533)

© 2018 Troeger C et al. *JAMA Pediatrics.*

Location	Deaths (95% UI)	Mortality rate per 100,000 (95% UI)	Incidence per 1,000 (95% UI)	Cases (95% UI)
Seychelles	0 (0-0)	0.4 (0.2-0.6)	351.8 (214.7-545.7)	2,955 (1,804-4,584)
Thailand	25 (15-39)	0.8 (0.5-1.3)	487.9 (366.5-664.4)	1,220,322 (916,601-1,661,770)
Timor-Leste	54 (27-98)	32.5 (16.1-59.0)	734.8 (482.6-1,108.8)	120,817 (79,346-182,321)
Vietnam	44 (25-73)	0.6 (0.3-1.0)	630.3 (422.7-909.1)	4,510,324 (3,024,761-6,505,488)
**Oceania**	**85 (44-153)**	**6.0 (3.1-10.8)**	**324.9 (208.3-487.1)**	**448,623 (287,666-672,555)**
American Samoa	0 (0-0)	0.5 (0.3-0.9)	276.8 (178.5-417.4)	2,069 (1,334-3,120)
Federated States of Micronesia	0 (0-0)	0.8 (0.3-1.5)	221.5 (140.9-332.2)	2,189 (1,393-3,283)
Fiji	4 (2-7)	7.0 (3.3-13.6)	311.2 (201.7-469.3)	16,951 (10,987-25,560)
Guam	0 (0-0)	0.2 (0.1-0.4)	283.8 (181.6-433.6)	4,960 (3,173-7,578)
Kiribati	1 (1-3)	11.0 (4.9-21.8)	343.2 (217.2-528.1)	4,665 (2,953-7,178)
Marshall Islands	0 (0-0)	1.1 (0.4-2.3)	262.8 (167.8-391.7)	2,582 (1,649-3,848)
Northern Mariana Islands	0 (0-0)	0.1 (0.0-0.1)	391.8 (244.9-619.4)	6,071 (3,794-9,598)
Papua New Guinea	72 (34-136)	6.7 (3.1-12.7)	334.9 (213.8-505.2)	348,845 (222,664-526,294)
Samoa	0 (0-0)	0.4 (0.1-0.8)	301.7 (193.9-458.9)	8,333 (5,355-12,674)
Solomon Islands	5 (2-9)	6.0 (3.0-10.7)	387.3 (254.0-575.1)	31,748 (20,827-47,144)
Tonga	0 (0-0)	1.0 (0.4-2.0)	283.5 (187.2-419.6)	3,880 (2,562-5,744)
Vanuatu	2 (1-4)	5.0 (2.3-9.9)	404.9 (266.9-596.1)	16,352 (10,778-24,071)
**Central Europe, Eastern Europe, and Central Asia**	**317 (212-456)**	**1.1 (0.8-1.6)**	**431.1 (296.2-605.9)**	**13,107,287 (9,005,945-18,424,868)**

© 2018 Troeger C et al. *JAMA Pediatrics.*

Location	Deaths (95% UI)	Mortality rate per 100,000 (95% UI)	Incidence per 1,000 (95% UI)	Cases (95% UI)
**Central Asia**	**237 (145-366)**	**2.5 (1.5-3.8)**	**172.5 (107.9-262.5)**	**1,899,833 (1,187,968-2,890,610)**
Armenia	1 (1-2)	0.5 (0.3-0.9)	233.5 (143.5-358.7)	52,505 (32,270-80,674)
Azerbaijan	27 (13-50)	2.8 (1.3-5.2)	303.6 (191.2-472.7)	281,222 (177,056-437,855)
Georgia	1 (1-3)	0.4 (0.2-0.8)	237.9 (144.1-368.0)	66,442 (40,234-102,773)
Kazakhstan	9 (5-15)	0.5 (0.3-0.8)	180.9 (112.3-276.0)	353,358 (219,333-539,129)
Kyrgyzstan	23 (15-34)	3.1 (2.0-4.5)	140.6 (89.2-212.8)	114,018 (72,375-172,623)
Mongolia	0 (0-1)	0.1 (0.1-0.2)	354.2 (230.4-528.2)	131,766 (85,733-196,511)
Tajikistan	143 (71-263)	12.6 (6.3-23.1)	304.4 (193.4-470.2)	334,272 (212,342-516,351)
Turkmenistan	17 (9-31)	3.0 (1.6-5.3)	353.6 (220.1-547.5)	189,520 (117,974-293,444)
Uzbekistan	15 (7-28)	0.4 (0.2-0.8)	78.7 (45.8-125.4)	378,433 (220,172-603,296)
**Central Europe**	**24 (19-32)**	**0.4 (0.3-0.6)**	**722.6 (501.7-1,027.8)**	**4,125,508 (2,864,684-5,868,045)**
Albania	0 (0-0)	0.1 (0.1-0.3)	337.0 (205.0-527.7)	58,485 (35,574-91,594)
Bosnia and Herzegovina	1 (0-1)	0.4 (0.2-0.6)	399.4 (253.4-593.6)	68,983 (43,757-102,511)
Bulgaria	2 (1-3)	0.5 (0.3-0.9)	508.9 (320.1-781.3)	172,970 (108,794-265,566)
Croatia	0 (0-0)	0.1 (0.1-0.2)	682.4 (404.2-1,046.0)	135,746 (80,404-208,086)
Czech Republic	1 (0-1)	0.1 (0.1-0.2)	586.9 (356.8-915.0)	332,219 (201,976-517,954)
Hungary	1 (0-1)	0.1 (0.1-0.2)	275.5 (167.4-435.1)	140,202 (85,187-221,431)
Macedonia	1 (0-1)	0.6 (0.3-1.2)	434.0 (263.5-670.6)	49,436 (30,014-76,382)

© 2018 Troeger C et al. *JAMA Pediatrics.*

Location	Deaths (95% UI)	Mortality rate per 100,000 (95% UI)	Incidence per 1,000 (95% UI)	Cases (95% UI)
Montenegro	0 (0-0)	0.2 (0.1-0.3)	297.3 (178.6-466.9)	10,995 (6,604-17,267)
Poland	2 (1-4)	0.1 (0.1-0.2)	860.8 (585.8-1,268.7)	1,710,463 (1,164,102-2,520,923)
Romania	15 (10-22)	1.7 (1.2-2.5)	1,186.8 (851.6-1,662.8)	945,983 (678,792-1,325,426)
Serbia	1 (1-2)	0.3 (0.2-0.5)	540.5 (343.6-784.4)	221,879 (141,021-321,989)
Slovakia	1 (0-1)	0.3 (0.1-0.5)	660.6 (384.2-1,053.4)	196,707 (114,399-313,656)
Slovenia	0 (0-0)	0.0 (0.0-0.1)	704.6 (408.7-1,100.3)	74,920 (43,458-116,990)
**Eastern Europe**	**55 (36-78)**	**0.4 (0.3-0.6)**	**520.4 (362.5-731.4)**	**7,122,181 (4,960,838-10,010,542)**
Belarus	1 (0-1)	0.1 (0.0-0.1)	400.4 (255.2-607.0)	239,056 (152,359-362,384)
Estonia	0 (0-0)	0.0 (0.0-0.1)	499.5 (324.4-687.6)	38,355 (24,908-52,796)
Latvia	0 (0-0)	0.1 (0.1-0.2)	581.7 (368.8-856.4)	75,203 (47,675-110,715)
Lithuania	0 (0-0)	0.1 (0.1-0.2)	748.5 (479.0-1,096.7)	132,183 (84,590-193,677)
Moldova	1 (1-2)	0.5 (0.3-0.8)	325.4 (210.5-482.7)	72,542 (46,916-107,600)
Russia	48 (31-71)	0.5 (0.3-0.8)	532.6 (374.7-749.4)	5,262,797 (3,703,129-7,405,344)
Ukraine	5 (2-10)	0.2 (0.1-0.4)	497.5 (318.4-730.1)	1,294,068 (828,264-1,899,123)
**High-income**	**134 (109-164)**	**0.2 (0.2-0.3)**	**118.8 (81.0-171.7)**	**6,903,717 (4,707,088-9,978,464)**
**High-income Asia Pacific**	**10 (8-13)**	**0.1 (0.1-0.2)**	**33.0 (20.7-49.9)**	**251,204 (157,464-379,899)**
Brunei	0 (0-0)	0.3 (0.2-0.4)	35.8 (22.3-54.1)	1,251 (781-1,891)
Japan	8 (6-10)	0.2 (0.1-0.2)	36.1 (22.2-54.5)	187,285 (115,095-283,218)

© 2018 Troeger C et al. *JAMA Pediatrics.*

Location	Deaths (95% UI)	Mortality rate per 100,000 (95% UI)	Incidence per 1,000 (95% UI)	Cases (95% UI)
South Korea	2 (1-3)	0.1 (0.1-0.1)	24.0 (14.7-37.9)	53,115 (32,501-83,835)
Singapore	0 (0-0)	0.1 (0.1-0.2)	53.6 (34.1-83.8)	9,503 (6,042-14,844)
Australasia	3 (2-4)	0.2 (0.1-0.2)	34.3 (21.7-52.3)	62,334 (39,402-94,978)
Australia	2 (2-3)	0.2 (0.1-0.2)	28.8 (18.0-44.2)	43,920 (27,465-67,236)
New Zealand	1 (0-1)	0.2 (0.2-0.3)	62.9 (40.0-98.3)	18,410 (11,703-28,755)
**Western Europe**	**50 (38-64)**	**0.2 (0.2-0.3)**	**219.2 (143.9-327.8)**	**4,866,571 (3,196,026-7,279,975)**
Andorra	0 (0-0)	0.2 (0.1-0.4)	232.9 (126.3-404.5)	696 (378-1,209)
Austria	1 (0-1)	0.1 (0.1-0.2)	270.5 (168.0-412.5)	106,729 (66,283-162,780)
Belgium	2 (1-2)	0.3 (0.2-0.4)	249.5 (152.1-386.4)	154,926 (94,413-239,902)
Cyprus	0 (0-1)	0.6 (0.3-1.1)	289.7 (158.6-505.6)	14,977 (8,200-26,140)
Denmark	2 (1-2)	0.5 (0.4-0.7)	453.3 (287.4-691.8)	129,552 (82,133-197,739)
England	5 (4-6)	0.1 (0.1-0.2)	144.9 (91.9-222.3)	460,317 (291,777-706,061)
Finland	0 (0-0)	0.1 (0.1-0.2)	736.0 (470.1-1,088.9)	214,607 (137,076-317,524)
France	18 (11-26)	0.5 (0.3-0.7)	261.9 (166.5-409.1)	1,022,477 (649,967-1,597,211)
Germany	6 (4-8)	0.2 (0.1-0.2)	255.2 (157.9-392.2)	886,893 (548,805-1,362,736)
Greece	0 (0-0)	0.0 (0.0-0.1)	104.6 (61.5-169.3)	49,740 (29,254-80,517)
Iceland	0 (0-0)	0.1 (0.1-0.2)	194.6 (118.5-306.5)	4,162 (2,535-6,554)
Ireland	0 (0-1)	0.1 (0.1-0.2)	111.7 (61.7-191.3)	41,774 (23,074-71,545)

© 2018 Troeger C et al. *JAMA Pediatrics.*

Location	Deaths (95% UI)	Mortality rate per 100,000 (95% UI)	Incidence per 1,000 (95% UI)	Cases (95% UI)
Israel	3 (2-4)	0.4 (0.3-0.5)	166.6 (117.3-246.5)	140,114 (98,615-207,280)
Italy	5 (3-7)	0.2 (0.1-0.3)	212.7 (151.6-286.7)	572,733 (408,164-771,831)
Luxembourg	0 (0-0)	0.3 (0.2-0.5)	240.1 (131.0-417.2)	6,992 (3,814-12,148)
Malta	0 (0-0)	0.1 (0.1-0.2)	95.4 (52.2-170.0)	2,028 (1,110-3,613)
Netherlands	2 (1-3)	0.2 (0.1-0.3)	428.5 (268.3-666.0)	372,773 (233,404-579,428)
Northern Ireland	1 (0-1)	0.4 (0.2-0.8)	198.6 (107.5-356.0)	23,551 (12,748-42,224)
Norway	0 (0-1)	0.1 (0.1-0.2)	212.9 (115.3-401.7)	60,285 (32,634-113,756)
Portugal	1 (1-2)	0.3 (0.2-0.5)	163.9 (98.8-253.6)	75,773 (45,686-117,262)
Scotland	1 (1-2)	0.3 (0.2-0.5)	193.5 (104.6-355.4)	52,705 (28,491-96,776)
Spain	1 (1-2)	0.1 (0.0-0.1)	78.0 (52.8-115.5)	185,335 (125,338-274,381)
Sweden	1 (0-1)	0.1 (0.1-0.2)	220.0 (137.7-339.9)	124,968 (78,254-193,095)
Switzerland	1 (1-2)	0.3 (0.2-0.4)	273.2 (172.4-417.0)	113,167 (71,437-172,737)
Wales	1 (0-1)	0.5 (0.3-0.8)	312.8 (170.2-560.4)	50,311 (27,373-90,124)
**Southern Latin America**	**33 (25-43)**	**0.7 (0.5-0.8)**	**224.0 (166.3-301.5)**	**1,121,370 (832,556-1,509,433)**
Argentina	28 (21-38)	0.8 (0.6-1.1)	236.7 (178.9-313.1)	839,195 (634,190-1,110,080)
Chile	2 (2-4)	0.2 (0.1-0.3)	211.3 (136.5-317.0)	257,959 (166,675-387,043)
Uruguay	2 (1-3)	0.8 (0.4-1.3)	100.7 (62.2-161.3)	24,036 (14,838-38,513)
**High-income North America**	**39 (29-50)**	**0.2 (0.1-0.2)**	**30.4 (19.2-45.2)**	**652,228 (412,958-970,517)**

© 2018 Troeger C et al. *JAMA Pediatrics.*

Location	Deaths (95% UI)	Mortality rate per 100,000 (95% UI)	Incidence per 1,000 (95% UI)	Cases (95% UI)
Canada	1 (1-2)	0.1 (0.0-0.1)	30.2 (17.9-48.5)	58,977 (34,983-94,549)
Greenland	0 (0-0)	0.3 (0.1-0.5)	40.9 (24.2-66.2)	141 (84-229)
United States	37 (28-48)	0.2 (0.1-0.2)	30.4 (19.2-44.8)	593,007 (375,338-875,292)
**Latin America and Caribbean**	**1,259 (976-1,595)**	**2.5 (2.0-3.2)**	**447.0 (307.3-614.1)**	**21,138,621 (14,532,864-29,044,618)**
**Caribbean**	**198 (116-328)**	**5.0 (2.9-8.2)**	**186.6 (111.4-299.3)**	**770,044 (459,648-1,234,977)**
Antigua and Barbuda	0 (0-0)	0.9 (0.4-1.7)	323.2 (171.0-585.1)	1,494 (790-2,705)
The Bahamas	0 (0-1)	0.7 (0.2-1.6)	273.1 (141.4-500.1)	8,720 (4,517-15,970)
Barbados	0 (0-0)	0.6 (0.2-1.3)	260.9 (135.9-483.0)	4,093 (2,132-7,576)
Belize	1 (0-2)	1.6 (0.6-3.6)	291.1 (159.0-523.2)	12,026 (6,567-21,613)
Bermuda	0 (0-0)	0.1 (0.1-0.2)	282.7 (141.9-529.7)	1,251 (628-2,345)
Cuba	3 (2-4)	0.4 (0.3-0.6)	206.4 (144.0-296.7)	127,330 (88,840-183,010)
Dominica	0 (0-0)	1.2 (0.6-2.3)	264.2 (138.8-485.6)	1,429 (751-2,625)
Dominican Republic	34 (17-61)	3.6 (1.9-6.5)	285.1 (146.4-535.8)	290,107 (148,973-545,229)
Grenada	0 (0-0)	0.7 (0.3-1.5)	255.5 (129.8-473.9)	3,048 (1,549-5,654)
Guyana	5 (3-9)	8.4 (4.8-13.7)	352.1 (226.7-524.5)	25,135 (16,185-37,439)
Haiti	146 (79-260)	9.6 (5.2-17.1)	119.7 (67.1-188.2)	181,081 (101,472-284,528)
Jamaica	4 (2-8)	1.4 (0.6-2.8)	175.1 (93.6-314.1)	46,862 (25,040-84,058)
Puerto Rico	1 (0-1)	0.3 (0.2-0.6)	185.7 (93.5-358.2)	44,635 (22,467-86,100)

© 2018 Troeger C et al. *JAMA Pediatrics.*

Location	Deaths (95% UI)	Mortality rate per 100,000 (95% UI)	Incidence per 1,000 (95% UI)	Cases (95% UI)
Saint Lucia	0 (0-0)	0.9 (0.3-2.0)	382.4 (203.5-688.8)	3,828 (2,037-6,894)
Saint Vincent and the Grenadines	0 (0-0)	1.7 (0.9-3.0)	230.7 (148.8-344.6)	2,198 (1,417-3,283)
Suriname	2 (1-4)	4.9 (2.4-8.9)	187.9 (117.2-293.7)	9,914 (6,181-15,497)
Trinidad and Tobago	1 (0-2)	0.9 (0.3-2.3)	146.1 (77.0-269.4)	12,978 (6,842-23,922)
Virgin Islands, U.S.	0 (0-0)	0.2 (0.1-0.4)	271.8 (139.3-509.7)	1,484 (761-2,784)
**Andean Latin America**	**80 (54-113)**	**1.2 (0.8-1.7)**	**256.1 (171.3-353.4)**	**1,679,785 (1,123,593-2,318,298)**
Bolivia	44 (25-73)	3.2 (1.8-5.3)	437.6 (294.3-640.0)	584,290 (392,939-854,572)
Ecuador	19 (11-30)	1.1 (0.6-1.7)	322.7 (195.1-482.5)	545,584 (329,857-815,742)
Peru	16 (9-25)	0.5 (0.3-0.7)	154.8 (98.8-221.6)	547,030 (349,305-783,048)
**Central Latin America**	**714 (538-930)**	**3.1 (2.4-4.1)**	**367.3 (241.6-533.6)**	**8,209,712 (5,400,527-11,925,432)**
Colombia	85 (55-125)	2.4 (1.5-3.5)	656.2 (424.6-966.3)	2,123,603 (1,374,212-3,127,391)
Costa Rica	3 (1-5)	0.8 (0.4-1.6)	441.4 (232.0-815.7)	143,184 (75,262-264,579)
El Salvador	16 (8-28)	2.9 (1.5-5.2)	550.2 (343.4-839.7)	257,963 (161,013-393,690)
Guatemala	139 (94-195)	7.0 (4.8-9.9)	390.5 (256.1-539.9)	747,833 (490,368-1,033,923)
Honduras	50 (29-80)	5.3 (3.1-8.5)	426.6 (275.0-603.1)	399,764 (257,735-565,116)
Mexico	282 (211-364)	2.4 (1.8-3.1)	203.9 (129.6-314.0)	2,378,537 (1,511,635-3,661,993)
Nicaragua	5 (3-9)	0.9 (0.5-1.4)	66.3 (31.1-115.3)	40,410 (18,937-70,302)
Panama	16 (9-29)	4.7 (2.5-8.3)	473.3 (247.8-858.2)	172,145 (90,108-312,113)

© 2018 Troeger C et al. *JAMA Pediatrics.*

Location	Deaths (95% UI)	Mortality rate per 100,000 (95% UI)	Incidence per 1,000 (95% UI)	Cases (95% UI)
Venezuela	119 (78-175)	4.1 (2.7-6.1)	694.2 (441.1-1,019.8)	1,965,004 (1,248,478-2,886,493)
**Tropical Latin America**	**267 (205-345)**	**1.7 (1.3-2.1)**	**740.1 (522.3-993.0)**	**10,553,252 (7,446,877-14,159,709)**
Brazil	259 (198-335)	1.7 (1.3-2.2)	734.9 (519.3-985.0)	10,248,349 (7,242,676-13,737,005)
Paraguay	9 (5-14)	1.4 (0.8-2.3)	976.9 (604.1-1,391.2)	305,549 (188,969-435,157)
**North Africa and Middle East**	**4,193 (2,714-6,422)**	**6.6 (4.3-10.2)**	**415.8 (267.4-608.8)**	**26,408,211 (16,981,918-38,665,090)**
Afghanistan	840 (412-1,520)	16.9 (8.3-30.7)	601.6 (332.9-1,017.3)	2,852,954 (1,578,830-4,824,054)
Algeria	43 (20-83)	0.9 (0.4-1.8)	252.3 (142.0-400.8)	1,284,098 (722,453-2,039,554)
Bahrain	0 (0-0)	0.1 (0.1-0.3)	251.8 (129.7-443.3)	24,282 (12,503-42,747)
Egypt	904 (463-1,558)	8.3 (4.2-14.3)	199.9 (123.9-297.1)	2,390,351 (1,481,493-3,552,297)
Iran	156 (55-363)	1.9 (0.7-4.5)	960.4 (623.8-1,423.3)	6,811,659 (4,424,130-10,094,515)
Iraq	312 (128-608)	4.1 (1.7-7.9)	375.9 (199.1-614.0)	2,783,505 (1,474,037-4,546,309)
Jordan	7 (4-11)	0.7 (0.4-1.1)	390.4 (251.3-571.9)	374,700 (241,231-548,908)
Kuwait	1 (0-1)	0.3 (0.1-0.5)	132.5 (69.1-226.4)	88,254 (46,017-150,762)
Lebanon	2 (1-4)	0.6 (0.3-1.3)	386.6 (214.3-637.3)	116,530 (64,594-192,120)
Libya	2 (1-4)	0.5 (0.2-0.9)	187.6 (106.8-296.9)	92,288 (52,519-146,079)
Morocco	235 (134-390)	10.3 (5.8-17.0)	475.9 (307.9-709.1)	1,068,125 (690,948-1,591,394)
Palestine	7 (3-12)	0.6 (0.3-1.1)	356.6 (190.9-595.7)	381,437 (204,199-637,220)
Oman	1 (1-3)	0.3 (0.2-0.6)	641.5 (406.6-956.8)	276,141 (175,029-411,882)

© 2018 Troeger C et al. *JAMA Pediatrics.*

Location	Deaths (95% UI)	Mortality rate per 100,000 (95% UI)	Incidence per 1,000 (95% UI)	Cases (95% UI)
Qatar	0 (0-0)	0.2 (0.1-0.4)	155.6 (83.1-280.9)	19,720 (10,530-35,585)
Saudi Arabia	7 (4-11)	0.3 (0.2-0.4)	224.4 (147.8-330.5)	572,180 (376,953-842,879)
Sudan	532 (207-1,173)	12.7 (4.9-28.0)	494.9 (344.7-693.7)	1,827,025 (1,272,500-2,561,104)
Syria	5 (2-10)	0.3 (0.1-0.6)	241.6 (127.1-414.8)	469,217 (246,940-805,803)
Tunisia	4 (2-7)	0.5 (0.3-0.9)	307.7 (235.3-406.8)	285,502 (218,380-377,533)
Turkey	39 (21-65)	0.6 (0.3-1.1)	257.1 (192.2-342.9)	1,602,391 (1,197,928-2,137,404)
United Arab Emirates	1 (0-2)	0.1 (0.0-0.3)	251.4 (130.8-434.7)	234,106 (121,806-404,747)
Yemen	1,093 (385-2,232)	23.6 (8.3-48.2)	659.3 (359.9-1,180.7)	2,978,163 (1,625,935-5,333,619)
**South Asia**	**13,396 (10,116-17,453)**	**8.7 (6.6-11.4)**	**220.1 (156.7-302.9)**	**35,169,025 (25,043,003-48,400,337)**
Bangladesh	782 (477-1,160)	5.5 (3.3-8.1)	414.4 (333.3-521.0)	6,181,744 (4,972,575-7,771,426)
Bhutan	3 (1-5)	3.6 (1.6-6.4)	898.7 (619.1-1,233.2)	67,965 (46,822-93,264)
India	10,282 (7,442-13,730)	9.2 (6.6-12.2)	194.7 (127.0-285.3)	22,612,577 (14,756,286-33,142,659)
Nepal	160 (84-284)	4.0 (2.1-7.1)	458.3 (377.1-554.5)	1,929,159 (1,587,098-2,333,911)
Pakistan	2,169 (1,411-3,123)	9.4 (6.1-13.5)	173.0 (117.3-241.5)	4,228,167 (2,865,976-5,902,830)
**Sub-Saharan Africa**	**104,733 (83,406-128,842)**	**66.9 (53.3-82.3)**	**742.6 (589.0-934.0)**	**117,303,716 (93,039,381-147,541,731)**
**Central Sub-Saharan Africa**	**15,617 (9,981-23,942)**	**75.1 (48.0-115.1)**	**1,353.4 (1,065.0-1,683.2)**	**28,733,279 (22,610,651-35,735,936)**
Angola	2,004 (1,114-3,280)	40.8 (22.7-66.9)	660.5 (476.2-949.4)	3,304,069 (2,381,788-4,749,019)
Central African Republic	1,232	166.0	1,263.2	999,223

© 2018 Troeger C et al. *JAMA Pediatrics.*

Location	Deaths (95% UI)	Mortality rate per 100,000 (95% UI)	Incidence per 1,000 (95% UI)	Cases (95% UI)
	(759-1,925)	(102.2-259.4)	(926.3-1,632.8)	(732,760-1,291,601)
Congo	273 (151-461)	36.6 (20.2-61.8)	944.9 (719.7-1,221.2)	704,108 (536,255-909,997)
Democratic Republic of the Congo	12,058 (6,601-19,978)	85.8 (47.0-142.1)	1,632.5 (1,290.7-2,012.3)	23,419,173 (18,515,882-28,867,977)
Equatorial Guinea	5 (2-10)	5.5 (2.5-10.6)	1,062.1 (645.2-1,590.1)	104,206 (63,301-156,012)
Gabon	44 (24-74)	18.1 (9.8-30.0)	789.7 (578.9-1,033.5)	196,075 (143,749-256,615)
**Eastern Sub-Saharan Africa**	**16,300 (13,040-19,805)**	**26.1 (20.8-31.7)**	**432.1 (330.6-560.5)**	**26,952,299 (20,623,853-34,965,179)**
Burundi	1,814 (1,084-2,927)	84.7 (50.6-136.6)	622.2 (392.9-924.5)	1,316,133 (831,106-1,955,556)
Comoros	18 (9-34)	19.6 (10.1-36.4)	534.7 (331.4-810.1)	51,972 (32,210-78,737)
Djibouti	19 (9-36)	11.1 (5.0-21.0)	473.1 (284.6-759.4)	59,294 (35,671-95,189)
Eritrea	289 (158-468)	36.3 (19.9-58.8)	523.0 (313.9-847.9)	392,488 (235,534-636,256)
Ethiopia	2,649 (1,716-3,738)	17.3 (11.2-24.5)	299.1 (233.9-372.1)	4,419,158 (3,455,906-5,496,306)
Kenya	1,404 (1,008-1,929)	21.4 (15.3-29.4)	343.2 (228.5-502.3)	2,260,592 (1,505,105-3,308,791)
Madagascar	1,718 (960-2,741)	44.2 (24.7-70.5)	358.5 (220.1-554.3)	1,384,649 (850,318-2,141,022)
Malawi	1,000 (599-1,541)	31.2 (18.7-48.1)	582.2 (375.7-857.7)	1,880,469 (1,213,479-2,770,214)
Mozambique	1,216 (741-1,841)	24.5 (14.9-37.1)	366.8 (282.0-481.4)	1,841,770 (1,416,213-2,417,525)
Rwanda	650 (411-985)	34.7 (21.9-52.6)	720.0 (571.8-913.5)	1,336,002 (1,060,959-1,694,960)
Somalia	518 (258-930)	39.2 (19.6-70.5)	636.2 (422.1-909.8)	752,300 (499,220-1,075,891)
South Sudan	913 (443-1,716)	31.9 (15.5-60.0)	719.9 (435.3-1,132.0)	2,146,188 (1,297,642-3,374,501)
Tanzania	1,224	13.6	277.0	2,601,351

© 2018 Troeger C et al. *JAMA Pediatrics.*

Location	Deaths (95% UI)	Mortality rate per 100,000 (95% UI)	Incidence per 1,000 (95% UI)	Cases (95% UI)
	(775-1,834)	(8.6-20.4)	(196.2-389.4)	(1,842,554-3,657,017)
Uganda	2,146 (1,442-3,070)	28.6 (19.2-40.8)	694.1 (536.4-883.6)	5,238,860 (4,048,229-6,669,040)
Zambia	722 (466-1,086)	25.4 (16.4-38.2)	454.8 (347.8-571.0)	1,283,627 (981,617-1,611,551)
**Southern Sub-Saharan Africa**	**1,514 (1,071-2,071)**	**17.6 (12.4-24.1)**	**237.3 (157.5-342.2)**	**2,150,738 (1,427,096-3,101,783)**
Botswana	11 (5-21)	4.3 (2.0-8.0)	77.6 (41.6-128.2)	21,110 (11,324-34,889)
Lesotho	141 (89-218)	54.7 (34.4-84.6)	299.1 (193.5-443.8)	75,510 (48,855-112,051)
Namibia	101 (53-163)	30.3 (16.0-48.9)	319.2 (196.7-477.0)	108,721 (67,011-162,469)
South Africa	319 (205-489)	6.4 (4.1-9.8)	220.5 (145.7-318.0)	1,191,643 (787,473-1,718,763)
Swaziland	75 (43-119)	36.0 (20.8-56.9)	244.6 (150.5-375.0)	50,008 (30,768-76,668)
Zimbabwe	866 (553-1,266)	34.2 (21.8-50.0)	271.2 (175.6-396.7)	702,165 (454,567-1,026,910)
**Western Sub-Saharan Africa**	**71,303 (53,105-91,870)**	**110.3 (82.2-142.2)**	**915.2 (731.2-1,151.4)**	**59,763,343 (47,747,505-75,187,780)**
Benin	1,806 (1,094-2,708)	94.5 (57.3-141.7)	572.8 (362.2-861.6)	1,118,918 (707,444-1,683,010)
Burkina Faso	2,699 (1,584-4,248)	84.5 (49.6-133.0)	1,208.2 (962.5-1,549.1)	3,888,875 (3,098,119-4,986,247)
Cameroon	1,961 (1,177-3,059)	50.6 (30.3-78.9)	904.3 (695.7-1,168.9)	3,556,390 (2,735,863-4,596,857)
Cape Verde	3 (2-5)	3.9 (2.3-6.0)	411.5 (251.3-639.9)	30,591 (18,683-47,569)
Chad	4,229 (2,693-6,486)	160.1 (101.9-245.6)	1,114.8 (695.8-1,610.1)	2,874,413 (1,793,880-4,151,413)
Cote d'Ivoire	2,596 (1,570-3,947)	72.9 (44.1-110.9)	636.3 (417.5-929.9)	2,224,796 (1,459,666-3,251,367)
The Gambia	37 (23-54)	10.0 (6.4-14.7)	176.8 (117.4-258.2)	64,914 (43,103-94,821)
Ghana	664	15.3	712.6	3,289,343

© 2018 Troeger C et al. *JAMA Pediatrics.*

Location	Deaths (95% UI)	Mortality rate per 100,000 (95% UI)	Incidence per 1,000 (95% UI)	Cases (95% UI)
	(410-1,025)	(9.4-23.6)	(575.1-877.9)	(2,654,688-4,052,310)
Guinea	850<br>(476-1,435)	41.8<br>(23.4-70.5)	762.8<br>(477.0-1,123.0)	1,526,681<br>(954,754-2,247,538)
Guinea-Bissau	195<br>(117-293)	63.1<br>(37.6-94.7)	799.7<br>(529.4-1,193.4)	244,354<br>(161,753-364,629)
Liberia	665<br>(401-1,024)	93.2<br>(56.3-143.6)	880.9<br>(552.2-1,340.8)	625,350<br>(391,992-951,882)
Mali	1,109<br>(694-1,668)	35.2<br>(22.0-53.0)	187.2<br>(130.4-268.4)	576,676<br>(401,781-826,975)
Mauritania	177<br>(110-271)	34.4<br>(21.3-52.6)	679.2<br>(438.0-995.6)	339,011<br>(218,583-496,915)
Niger	4,160<br>(2,654-6,002)	112.9<br>(72.1-162.9)	716.3<br>(573.0-908.9)	2,656,642<br>(2,125,232-3,371,236)
Nigeria	47,683<br>(31,726-66,272)	161.5<br>(107.4-224.4)	1,116.0<br>(913.6-1,386.3)	33,510,145<br>(27,432,439-41,628,263)
Sao Tome and Principe	5<br>(3-10)	16.0<br>(7.6-28.6)	601.8<br>(375.1-918.0)	20,796<br>(12,962-31,722)
Senegal	903<br>(575-1,317)	35.5<br>(22.6-51.8)	613.0<br>(421.6-851.2)	1,571,014<br>(1,080,596-2,181,663)
Sierra Leone	1,122<br>(676-1,782)	107.9<br>(65.0-171.4)	623.1<br>(396.8-983.2)	648,546<br>(412,969-1,023,301)
Togo	439<br>(257-694)	40.1<br>(23.5-63.4)	885.9<br>(697.8-1,118.2)	967,975<br>(762,380-1,221,812)

© 2018 Troeger C et al. *JAMA Pediatrics.*

eTable 5. Deaths averted due to the rotavirus vaccine and remaining avertable deaths if vaccine coverage were 100% in 2016 by GBD region and country.

Location	Deaths averted	Avertable deaths
**Global**	**28844 (14600-46750)**	**83158 (36954-168037)**
**High-income**	**67 (44-94)**	**69 (32-135)**
**High-income North America**	**31 (21-42)**	**13 (6-23)**
Canada	0 (0-0)	1 (0-2)
Greenland	0 (0-0)	0 (0-0)
United States	31 (21-41)	12 (6-21)
**Australasia**	**4 (2-6)**	**1 (0-2)**
Australia	3 (2-6)	0 (0-1)
New Zealand	0 (0-0)	0 (0-1)
**High-income Asia Pacific**	**0 (0-0)**	**8 (4-14)**
Brunei	0 (0-0)	0 (0-0)
Japan	0 (0-0)	6 (3-10)
Singapore	0 (0-0)	0 (0-0)
South Korea	0 (0-0)	2 (1-3)
**Western Europe**	**20 (12-34)**	**31 (14-64)**
Andorra	0 (0-0)	0 (0-0)
Austria	1 (0-1)	0 (0-0)
Belgium	3 (1-5)	0 (0-0)
Cyprus	0 (0-0)	0 (0-1)
Denmark	0 (0-0)	1 (1-2)
Finland	0 (0-1)	0 (0-0)
France	0 (0-0)	14 (6-27)
Germany	2 (1-3)	3 (1-7)
Greece	0 (0-0)	0 (0-0)

© 2018 Troeger C et al. *JAMA Pediatrics.*

Location	Deaths averted	Avertable deaths
Iceland	0<br>(0-0)	0<br>(0-0)
Ireland	0<br>(0-0)	0<br>(0-1)
Israel	4<br>(2-6)	1<br>(0-2)
Italy	0<br>(0-0)	3<br>(2-7)
Luxembourg	0<br>(0-0)	0<br>(0-0)
Malta	0<br>(0-0)	0<br>(0-0)
Netherlands	0<br>(0-0)	2<br>(1-3)
Norway	0<br>(0-0)	0<br>(0-1)
Portugal	0<br>(0-0)	1<br>(0-2)
Spain	0<br>(0-0)	1<br>(0-2)
Sweden	0<br>(0-0)	0<br>(0-1)
Switzerland	0<br>(0-0)	1<br>(0-2)
England	4<br>(3-5)	1<br>(1-2)
Northern Ireland	1<br>(0-2)	0<br>(0-0)
Scotland	1<br>(0-3)	0<br>(0-1)
Wales	1<br>(0-2)	0<br>(0-1)
**Southern Latin America**	**12<br>(10-12)**	**16<br>(7-33)**
Argentina	12<br>(10-12)	13<br>(6-26)
Chile	0<br>(0-0)	2<br>(1-4)
Uruguay	0<br>(0-0)	1<br>(1-3)
**Central Europe, Eastern Europe, and Central Asia**	**175<br>(61-384)**	**161<br>(61-405)**
**Eastern Europe**	**1<br>(1-1)**	**41<br>(20-84)**
Belarus	0<br>(0-0)	0<br>(0-1)
Estonia	0<br>(0-0)	0<br>(0-0)
Latvia	0<br>(0-0)	0<br>(0-0)
Lithuania	0<br>(0-0)	0<br>(0-0)
Moldova	1	0

© 2018 Troeger C et al. *JAMA Pediatrics.*

Location	Deaths averted	Avertable deaths
	(0-1)	(0-1)
Ukraine	0<br>(0-0)	4<br>(1-10)
**Central Europe**	**0<br>(0-0)**	**19<br>(9-37)**
Albania	0<br>(0-0)	0<br>(0-1)
Bosnia and Herzegovina	0<br>(0-0)	0<br>(0-1)
Bulgaria	0<br>(0-0)	1<br>(0-3)
Croatia	0<br>(0-0)	0<br>(0-0)
Czech Republic	0<br>(0-0)	1<br>(0-1)
Hungary	0<br>(0-0)	0<br>(0-1)
Macedonia	0<br>(0-0)	1<br>(0-1)
Montenegro	0<br>(0-0)	0<br>(0-0)
Poland	0<br>(0-0)	2<br>(1-4)
Romania	0<br>(0-0)	12<br>(6-20)
Russian	0<br>(0-0)	37<br>(18-72)
Serbia	0<br>(0-0)	1<br>(0-2)
Slovakia	0<br>(0-0)	1<br>(0-1)
Slovenia	0<br>(0-0)	0<br>(0-0)
**Central Asia**	**174<br>(60-383)**	**101<br>(32-284)**
Armenia	2<br>(1-4)	0<br>(0-0)
Azerbaijan	0<br>(0-0)	20<br>(6-55)
Georgia	1<br>(0-2)	1<br>(0-1)
Kazakhstan	0<br>(0-0)	7<br>(3-16)
Kyrgyzstan	0<br>(0-0)	18<br>(8-36)
Mongolia	0<br>(0-0)	0<br>(0-1)
Tajikistan	130<br>(48-261)	41<br>(10-140)
Turkmenistan	0<br>(0-0)	13<br>(5-32)
Uzbekistan	41<br>(11-115)	0<br>(0-2)

© 2018 Troeger C et al. *JAMA Pediatrics.*

Location	Deaths averted	Avertable deaths
**Latin America and Caribbean**	**1618 (875-2597)**	**308 (113-782)**
**Central Latin America**	**867 (459-1382)**	**176 (73-405)**
Colombia	171 (79-286)	8 (2-27)
Costa Rica	0 (0-0)	2 (1-6)
El Salvador	19 (8-34)	3 (1-11)
Guatemala	260 (129-422)	16 (5-45)
Honduras	83 (34-148)	7 (2-24)
Mexico	244 (159-347)	84 (42-158)
Nicaragua	15 (4-37)	0 (0-1)
Panama	11 (5-21)	6 (2-19)
Venezuela	63 (40-87)	49 (19-115)
**Andean Latin America**	**102 (45-177)**	**16 (4-53)**
Bolivia	52 (22-90)	10 (2-32)
Ecuador	33 (15-58)	2 (1-9)
Peru	17 (8-29)	4 (1-12)
**Caribbean**	**95 (43-169)**	**90 (25-269)**
Antigua and Barbuda	0 (0-0)	0 (0-0)
The Bahamas	0 (0-0)	0 (0-1)
Barbados	0 (0-0)	0 (0-0)
Belize	0 (0-0)	1 (0-2)
Bermuda	0 (0-0)	0 (0-0)
Cuba	0 (0-0)	2 (1-4)
Dominica	0 (0-0)	0 (0-0)
Dominican Republic	15 (6-29)	16 (4-52)
Grenada	0 (0-0)	0 (0-0)
Guyana	10 (4-19)	1 (0-2)
Haiti	69	65

© 2018 Troeger C et al. *JAMA Pediatrics.*

Location	Deaths averted	Avertable deaths
	(32-118)	(18-191)
Jamaica	0<br>(0-0)	3<br>(1-10)
Puerto Rico	1<br>(0-2)	0<br>(0-0)
Saint Lucia	0<br>(0-0)	0<br>(0-0)
Saint Vincent and the Grenadines	0<br>(0-0)	0<br>(0-0)
Suriname	0<br>(0-0)	2<br>(1-4)
Trinidad and Tobago	0<br>(0-0)	0<br>(0-2)
Virgin Islands, U.S.	0<br>(0-0)	0<br>(0-0)
**Tropical Latin America**	**554<br>(327-869)**	**25<br>(11-55)**
Brazil	549<br>(325-861)	22<br>(10-45)
Paraguay	5<br>(3-8)	3<br>(1-10)
**Southeast Asia, East Asia, and Oceania**	**1410<br>(855-2162)**	**2791<br>(1307-5776)**
**East Asia**	**864<br>(511-1379)**	**190<br>(72-462)**
China	864<br>(511-1379)	56<br>(28-110)
North Korea	0<br>(0-0)	132<br>(43-348)
Taiwan	0<br>(0-0)	2<br>(1-3)
**Southeast Asia**	**542<br>(342-775)**	**2539<br>(1217-5139)**
Cambodia	0<br>(0-0)	73<br>(27-179)
Indonesia	0<br>(0-0)	1523<br>(883-2523)
Laos	0<br>(0-0)	252<br>(72-696)
Malaysia	0<br>(0-0)	6<br>(2-13)
Maldives	0<br>(0-0)	0<br>(0-0)
Mauritius	1<br>(0-1)	0<br>(0-1)
Myanmar	0<br>(0-0)	247<br>(75-672)
Philippines	541<br>(341-774)	341<br>(122-825)
Sri Lanka	0<br>(0-0)	2<br>(1-6)
Seychelles	0<br>(0-0)	0<br>(0-0)

© 2018 Troeger C et al. *JAMA Pediatrics.*

Location	Deaths averted	Avertable deaths
Thailand	0<br>(0-0)	19<br>(8-39)
Timor-Leste	0<br>(0-0)	41<br>(14-105)
Vietnam	0<br>(0-0)	34<br>(13-79)
**Oceania**	**5<br>(2-8)**	**63<br>(18-175)**
American Samoa	0<br>(0-0)	0<br>(0-0)
Federated States of Micronesia	0<br>(0-0)	0<br>(0-0)
Fiji	4<br>(1-7)	1<br>(0-3)
Guam	0<br>(0-0)	0<br>(0-0)
Kiribati	1<br>(0-2)	1<br>(0-2)
Marshall Islands	0<br>(0-0)	0<br>(0-0)
Northern Mariana Islands	0<br>(0-0)	0<br>(0-0)
Papua New Guinea	0<br>(0-0)	55<br>(16-154)
Samoa	0<br>(0-0)	0<br>(0-0)
Solomon Islands	0<br>(0-0)	4<br>(1-10)
Tonga	0<br>(0-0)	0<br>(0-0)
Vanuatu	0<br>(0-0)	2<br>(0-4)
**North Africa and Middle East**	**1220<br>(403-2638)**	**2502<br>(655-7481)**
Afghanistan	0<br>(0-0)	642<br>(182-1792)
Algeria	0<br>(0-0)	33<br>(9-101)
Bahrain	0<br>(0-1)	0<br>(0-0)
Egypt	0<br>(0-0)	690<br>(238-1677)
Iran	0<br>(0-0)	119<br>(30-375)
Iraq	93<br>(27-199)	167<br>(31-554)
Jordan	8<br>(4-12)	2<br>(0-5)
Kuwait	0<br>(0-0)	1<br>(0-2)
Lebanon	0<br>(0-0)	2<br>(0-5)
Libya	5	0

© 2018 Troeger C et al. *JAMA Pediatrics.*

Location	Deaths averted	Avertable deaths
	(1-13)	(0-1)
Morocco	426 (167-863)	28 (7-94)
Palestine	0 (0-0)	5 (1-15)
Oman	0 (0-0)	1 (0-3)
Qatar	0 (0-1)	0 (0-0)
Saudi Arabia	12 (5-24)	1 (0-2)
Sudan	299 (92-680)	217 (43-764)
Syria	0 (0-0)	4 (1-12)
Tunisia	0 (0-0)	3 (1-7)
Turkey	0 (0-0)	30 (11-66)
United Arab Emirates	2 (0-5)	0 (0-1)
Yemen	373 (106-839)	558 (99-2004)
**South Asia**	**147 (94-208)**	**10093 (5172-18121)**
Bangladesh	0 (0-0)	598 (268-1177)
Bhutan	0 (0-0)	2 (1-6)
India	147 (94-208)	7712 (4169-13324)
Nepal	0 (0-0)	122 (42-290)
Pakistan	0 (0-0)	1659 (692-3324)
**Sub-Saharan Africa**	**24206 (12268-38666)**	**67234 (29614-135336)**
**Southern Sub-Saharan Africa**	**1686 (833-2589)**	**409 (128-1113)**
Botswana	23 (7-46)	1 (0-6)
Lesotho	0 (0-0)	108 (46-224)
Namibia	69 (34-106)	36 (9-101)
South Africa	316 (164-512)	85 (28-221)
Swaziland	68 (44-61)	22 (4-76)
Zimbabwe	1210 (584-1864)	157 (41-485)
**Western Sub-Saharan Africa**	**9827 (5285-14768)**	**48660 (22281-94596)**

© 2018 Troeger C et al. *JAMA Pediatrics.*

Location	Deaths averted	Avertable deaths
Benin	0<br>(0-0)	1380<br>(544-2982)
Burkina Faso	2797<br>(1492-4070)	689<br>(206-1973)
Cameroon	1202<br>(646-1812)	753<br>(265-1837)
Cape Verde	0<br>(0-0)	2<br>(1-5)
Chad	0<br>(0-0)	3235<br>(1309-6942)
Cote d'Ivoire	922<br>(486-1463)	1301<br>(429-3218)
The Gambia	33<br>(17-51)	11<br>(3-29)
Ghana	877<br>(454-1335)	130<br>(39-366)
Guinea	0<br>(0-0)	649<br>(218-1642)
Guinea-Bissau	118<br>(60-188)	76<br>(24-194)
Liberia	117<br>(64-183)	408<br>(149-952)
Mali	208<br>(120-310)	671<br>(251-1524)
Mauritania	35<br>(19-58)	106<br>(39-251)
Niger	1740<br>(1044-2360)	1955<br>(715-4356)
Nigeria	0<br>(0-0)	36428<br>(17817-66032)
Sao Tome and Principe	3<br>(1-5)	2<br>(1-7)
Senegal	852<br>(426-1442)	252<br>(80-655)
Sierra Leone	667<br>(321-1091)	439<br>(134-1197)
Togo	256<br>(136-401)	174<br>(57-432)
**Eastern Sub-Saharan Africa**	**11580<br>(5538-19647)**	**6976<br>(2673-16128)**
Burundi	2544<br>(1097-4348)	327<br>(76-1114)
Comoros	0<br>(0-0)	14<br>(4-38)
Djibouti	8<br>(3-16)	9<br>(2-29)
Eritrea	673<br>(221-1506)	19<br>(3-77)
Ethiopia	584<br>(424-711)	1545<br>(661-2998)
Kenya	1103<br>(654-1682)	455<br>(208-916)
Madagascar	1119	636

© 2018 Troeger C et al. *JAMA Pediatrics.*

Location	Deaths averted	Avertable deaths
	(512-1885)	(177-1797)
Malawi	1394<br>(605-2554)	181<br>(52-532)
Mozambique	545<br>(305-787)	553<br>(198-1277)
Rwanda	1358<br>(566-2628)	57<br>(14-178)
Somalia	0<br>(0-0)	396<br>(125-1076)
South Sudan	0<br>(0-0)	700<br>(205-1921)
Tanzania	1534<br>(762-2465)	255<br>(68-755)
Uganda	0<br>(0-0)	1639<br>(817-2937)
Zambia	719<br>(388-1062)	192<br>(63-483)
**Central Sub-Saharan Africa**	**1113**<br>**(613-1663)**	**11188**<br>**(4533-23499)**
Angola	976<br>(536-1466)	874<br>(256-2315)
Central African Republic	0<br>(0-0)	942<br>(414-1889)
Congo	137<br>(77-197)	117<br>(38-291)
Democratic Republic of the Congo	0<br>(0-0)	9217<br>(3811-18915)
Equatorial Guinea	0<br>(0-0)	4<br>(1-12)
Gabon	0<br>(0-0)	34<br>(12-77)

© 2018 Troeger C et al. *JAMA Pediatrics.*

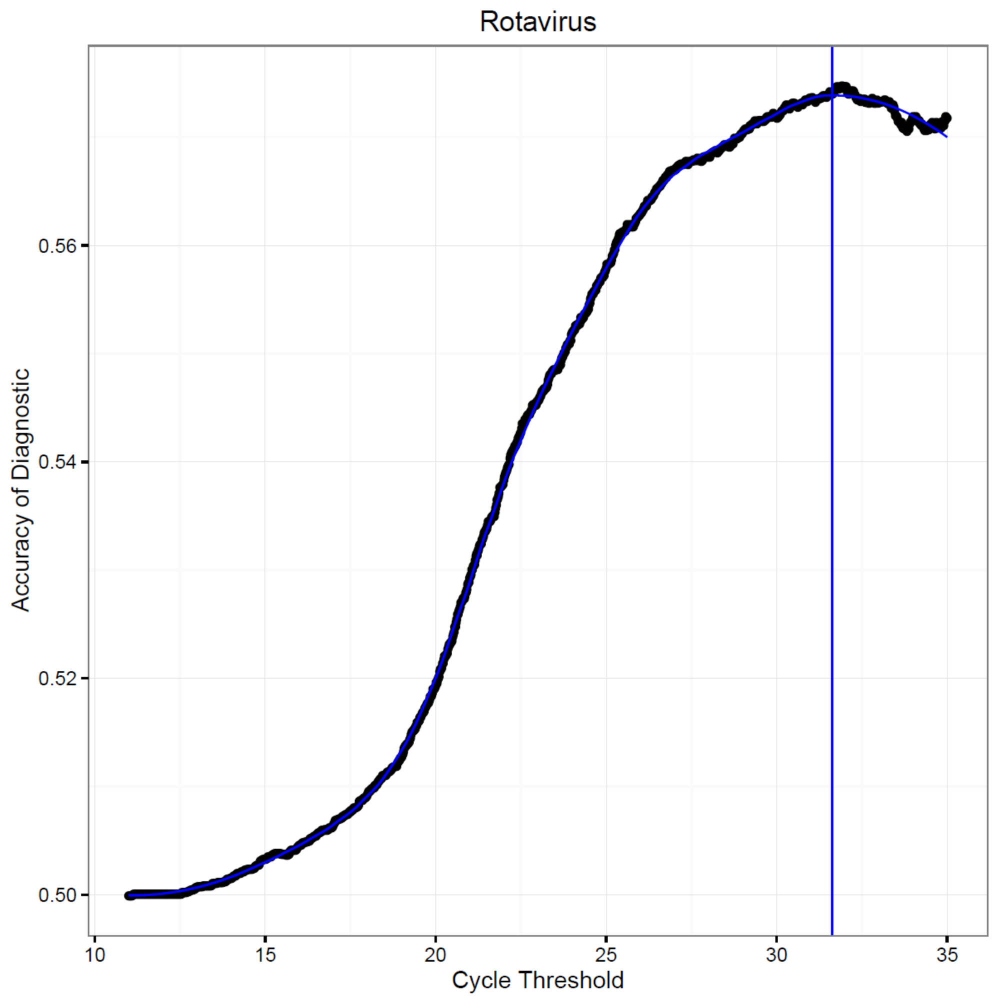

© 2018 Troeger C et al. *JAMA Pediatrics.*

eFigure 1. Data coverage maps.

A) Shows the number of unique sources providing data in the rotavirus model and B) shows the number of diarrhea samples from the data sources (i.e. the population sample size of the data). The data shown detail the proportion of diarrhea episodes where rotavirus is present.

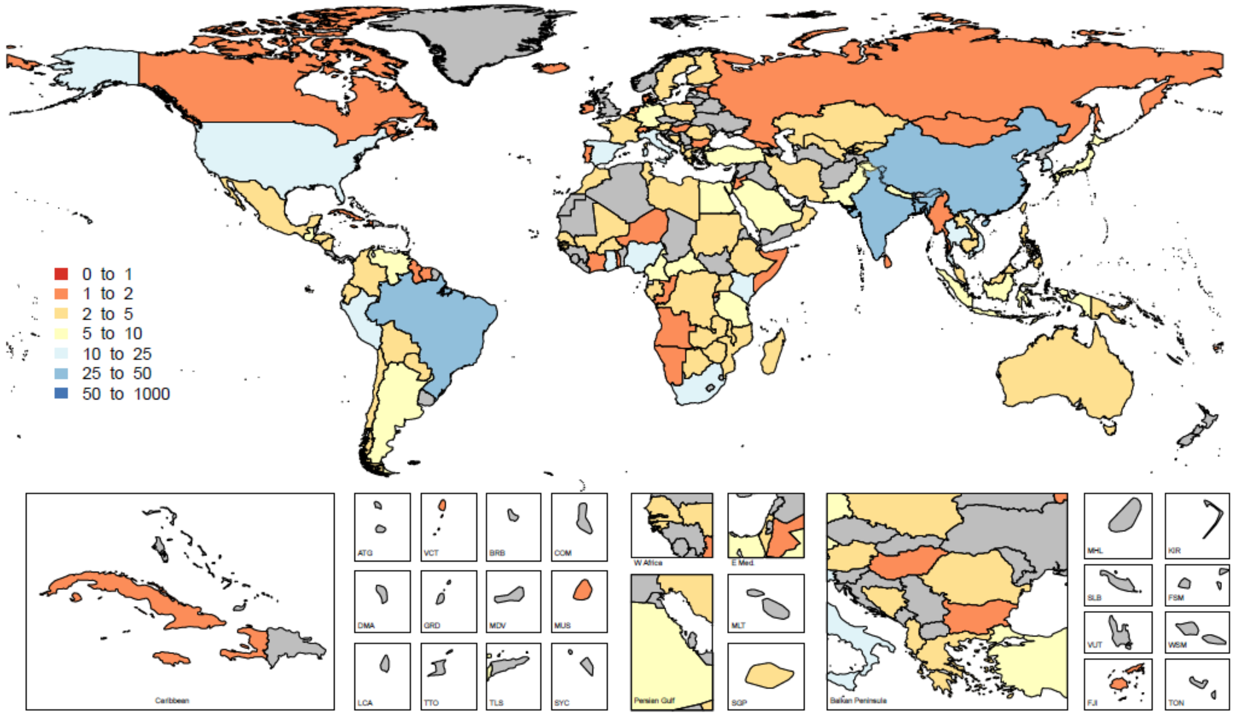

B)

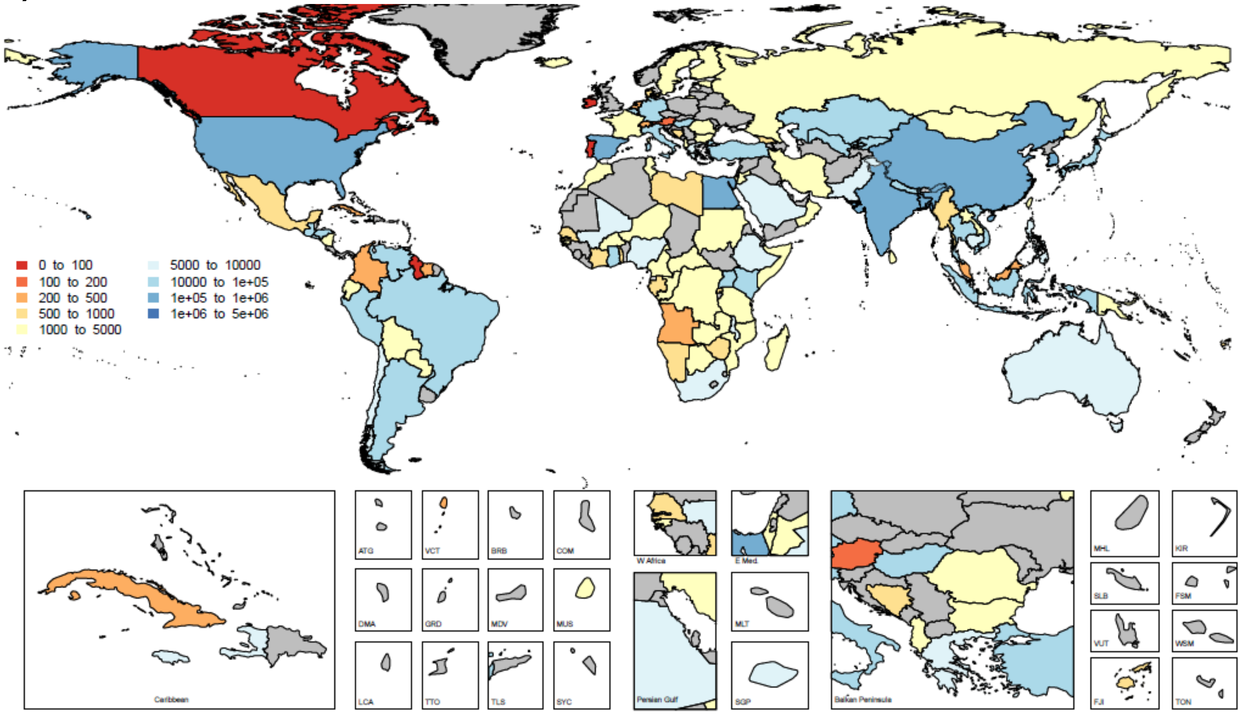

© 2018 Troeger C et al. *JAMA Pediatrics.*

eFigure 2. Diagnostic ability to discriminate cases and controls of qPCR.

The modeling strategy requires that the continuous qPCR test results are dichotomized into positive and negative results so that the odds represent the strength of association between rotavirus and diarrhea. The ability to discriminate a stool sample as either a case or control, termed the diagnostic accuracy, of the quantitative PCR (qPCR) array for rotavirus is shown where the relative quantity of rotavirus in a stool sample decreases from left to right. The accuracy is the proportion of stool samples that are correctly classified as case or control. We defined a cutoff point for the presence of rotavirus as the point where the diagnostic accuracy is maximized (blue line).

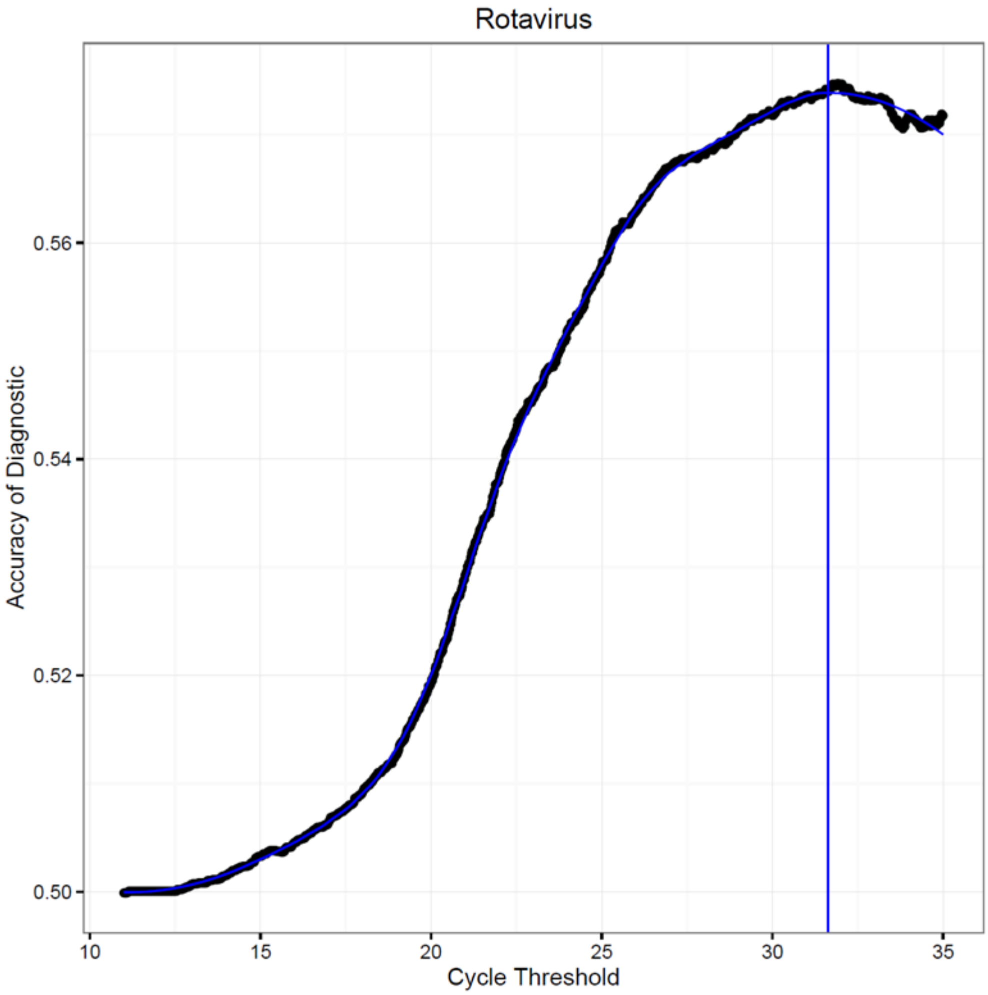

© 2018 Troeger C et al. *JAMA Pediatrics.*

eFigure 3. Modeled rotavirus vaccine coverage among children under 5 in 2016.

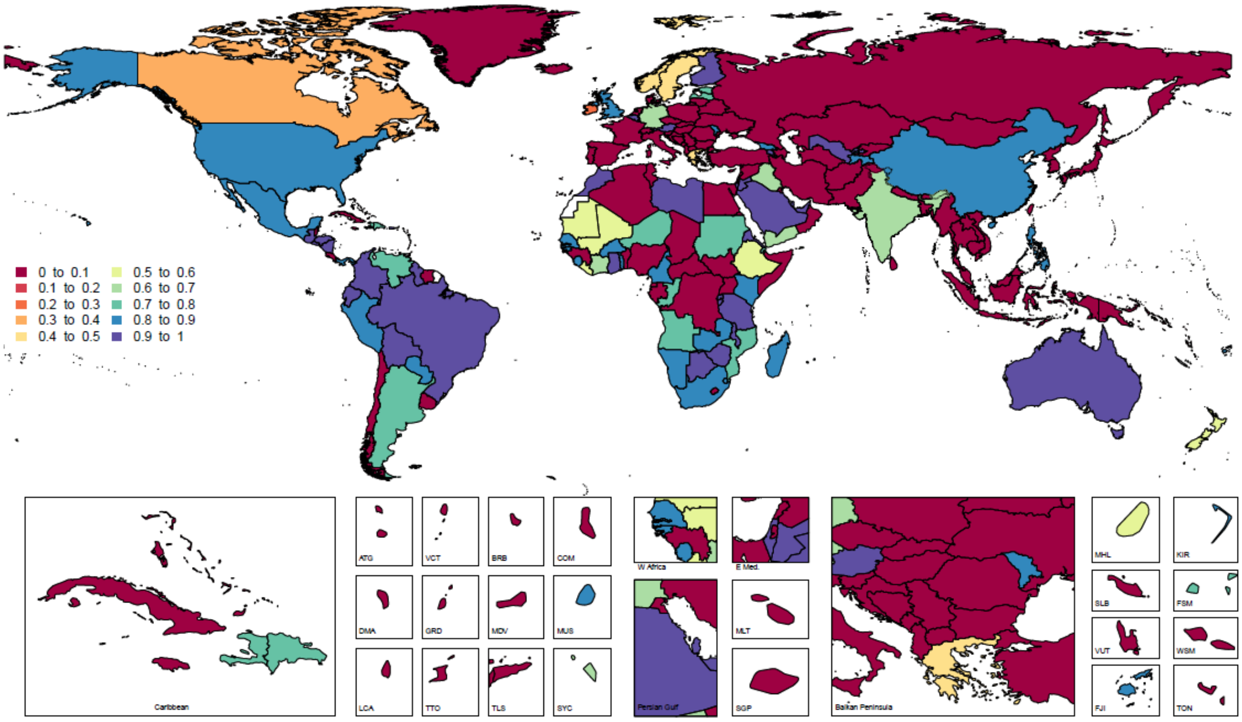

eFigure 4. Modeled number of under 5 deaths averted due to rotavirus vaccine in 2016.

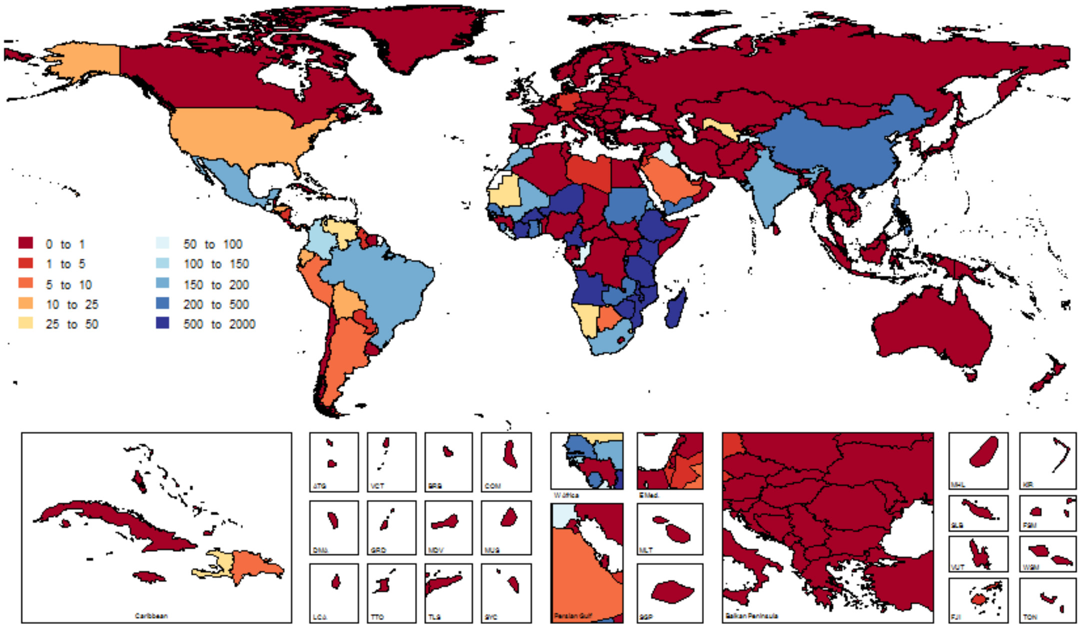

© 2018 Troeger C et al. *JAMA Pediatrics.*

eFigure 5. A comparison of the number of deaths and PAFs due to rotaviral diarrhea in GBD 2015 and GBD 2016 in all ages and children under-5.

The axes are on a log10 scale and locations are labeled with the iso3 code (see Supplementary Table 2) and colored to represent GBD regions.

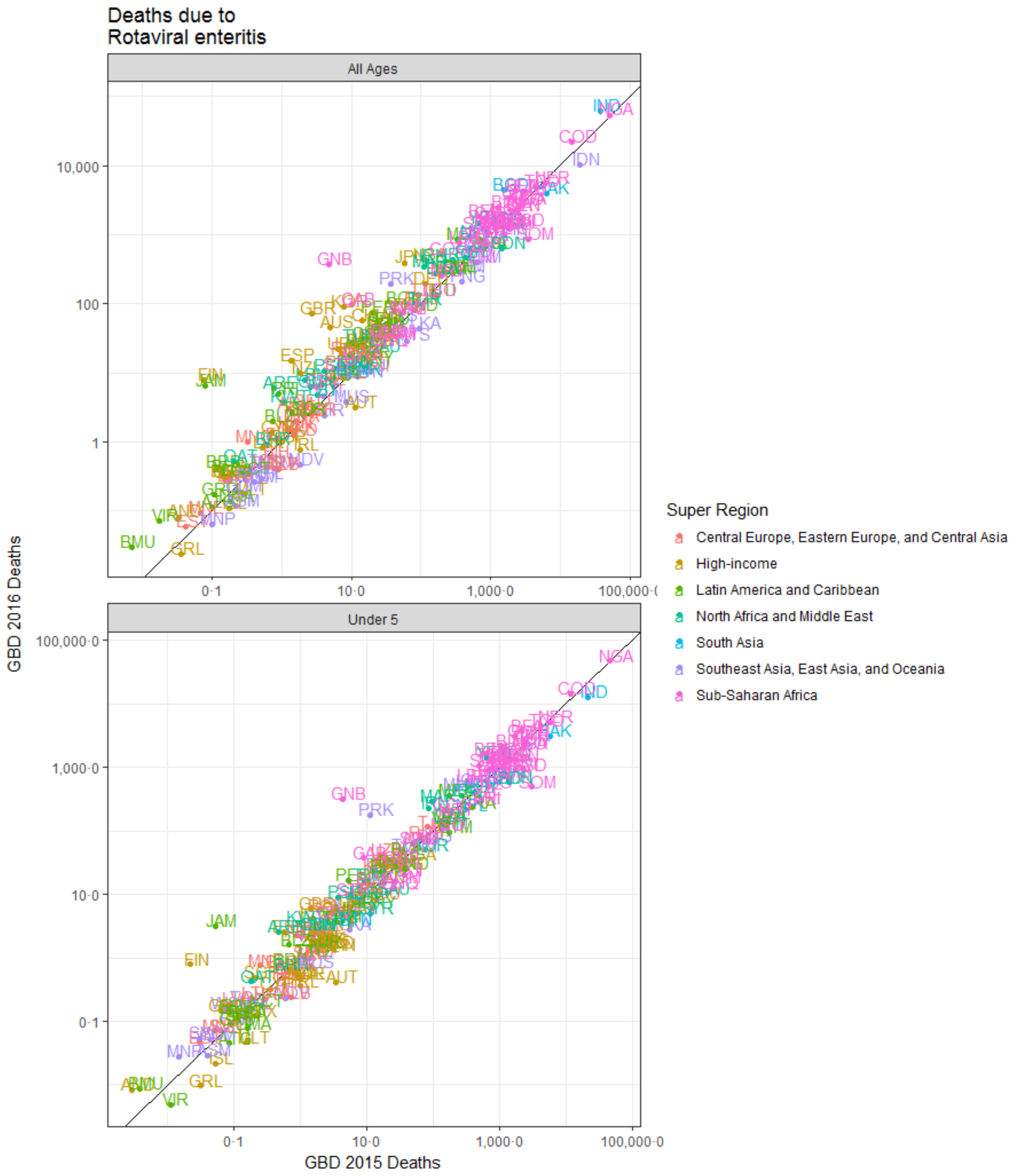

© 2018 Troeger C et al. *JAMA Pediatrics.*

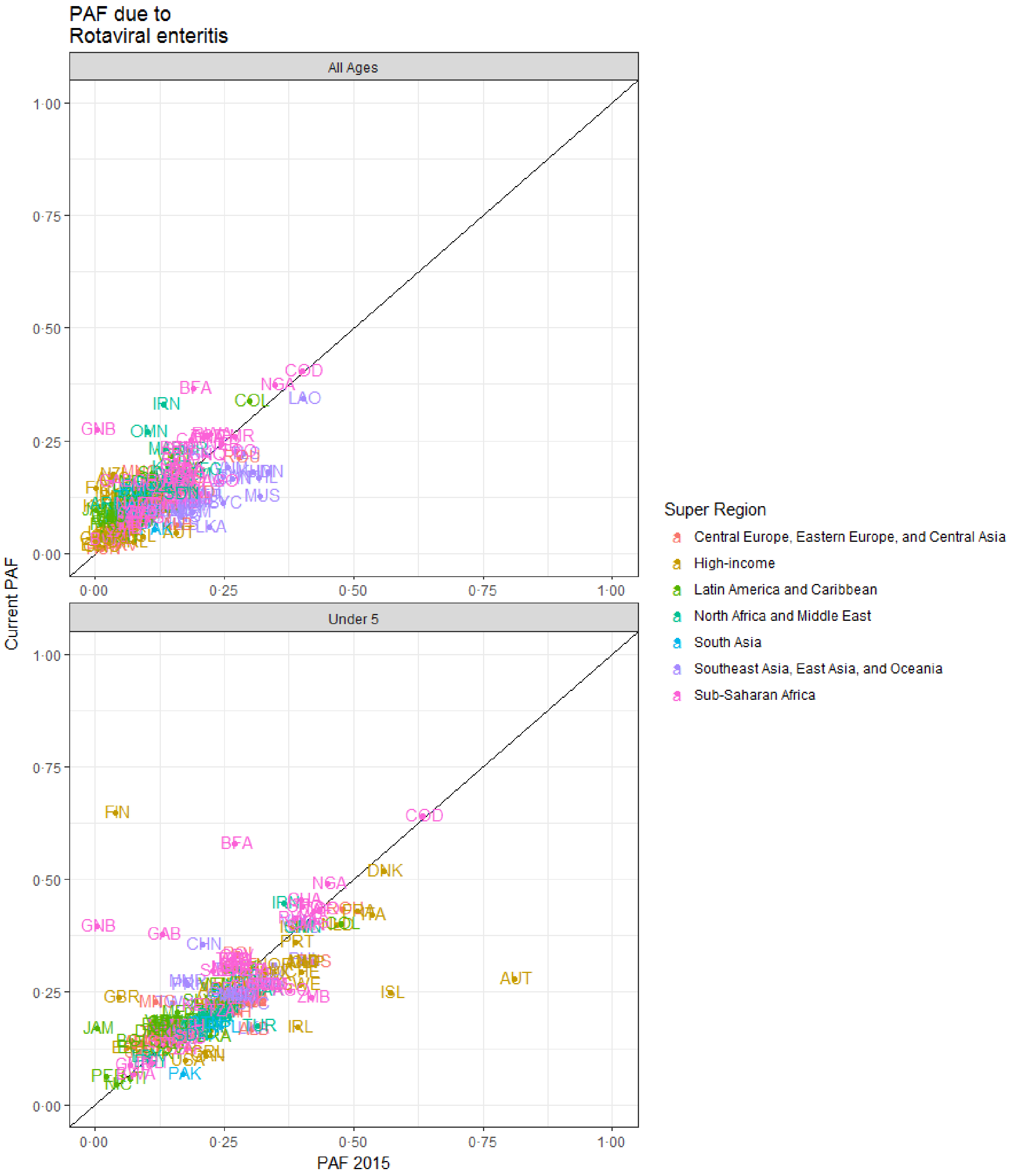

© 2018 Troeger C et al. *JAMA Pediatrics.*

eFigure 6. Visualizing uncertainty around PAF estimates.
These plots show the standard error of the distribution of PAF draws by region and rotavirus burden.

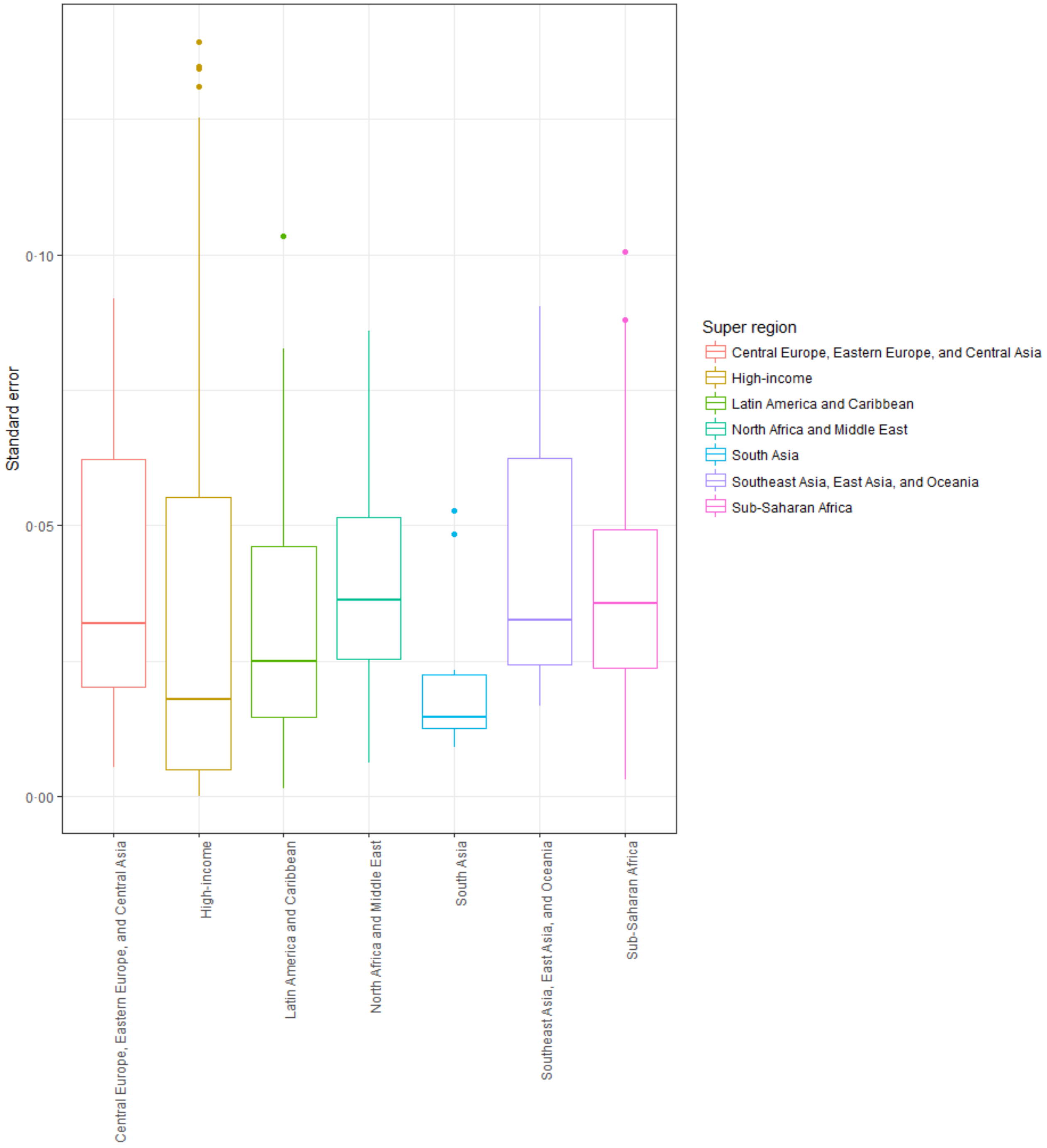

© 2018 Troeger C et al. *JAMA Pediatrics.*

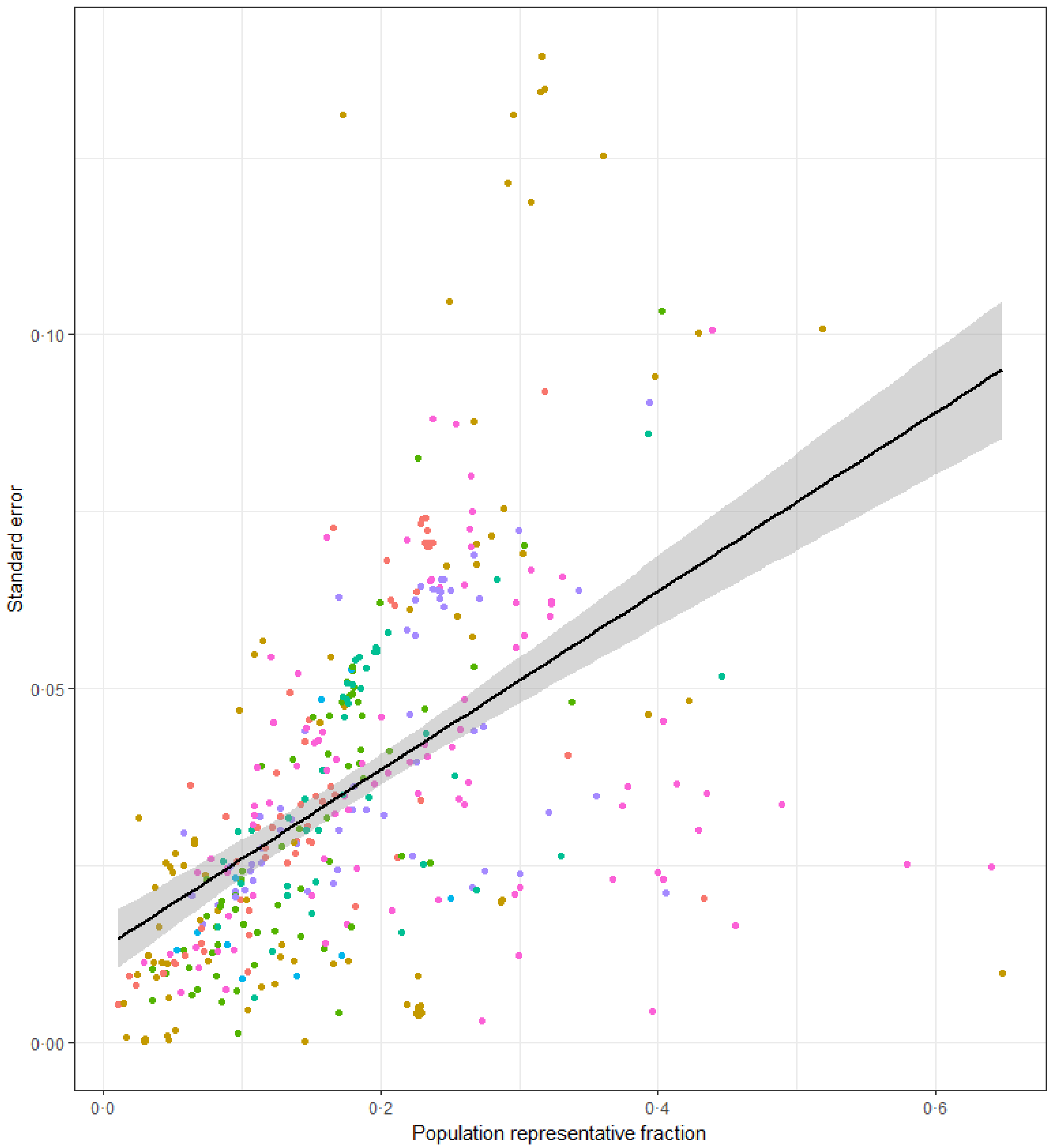

© 2018 Troeger C et al. *JAMA Pediatrics.*
